# HK-DeepIV: heat-kernel geometric diagnostics and early-warning signals for curvature-induced interference in financial correlation networks

**DOI:** 10.3389/fdata.2026.1925931

**Published:** 2026-09-03

**Authors:** Ntebogang Dinah Moroke

**Affiliations:** Department of Statistics and Operations Research, North-West University, Mafikeng Campus, Potchefstroom, South Africa

**Keywords:** causal inference, deep instrumental variables, Fiedler eigenvalue, financial networks, heat-kernel diffusion, Infrastructure resilience, Ollivier-Ricci curvature, systemic risk

## Abstract

Financial correlation networks under infrastructure shocks undergo curvature-driven interference that standard estimators cannot detect. This study introduces Heat-Kernel Deep Instrumental Variables (HK-DeepIV), a geometric diagnostic and early-warning framework modeling shock propagation as heat diffusion on the Riemannian manifold of Johannesburg Stock Exchange (JSE) asset-return correlations. Three formal results underpin the architecture: a non-parametric identification result for the projection of the structural function onto the leading heat-kernel eigenfunction (*k* = 1; a scalar instrument can identify at most one linear combination of the eigenfunctions, and no claim is made beyond that projection); double robustness via Neyman orthogonality; and Corollary 3.2, showing that unit-level distinguishability collapses exponentially in diffusion time whenever Ollivier-Ricci curvature is positive; a purely geometric statement providing the basis for the fragility diagnostics developed here. Applied to 60 JSE tickers (2,832 trading days, 2015–2025) with Eskom load-shedding as the treatment (2022–2025), the trained model produces an ATE of +383 bp, reported as an overfitting artifact. Five independent baselines converge on smaller, mostly negative estimates. The most credibly conditioned conditional-association estimate is double machine learning [−20.75 bp, heteroskedasticity-and-autocorrelation-consistent (HAC)-corrected 95% CI [−51.62, 10.12] bp, *p* = 0.188], which is not significant at conventional levels; consistent with the instrument exogeneity caveat (5-day lagged return balance test, *p* = 0.007) and the descriptive framing of all estimates. The instrument [48-h-ahead Eskom stage forecast, Corr(*Z*_*t*_, *D*_*t*_)≈0.71, first-stage *F* = 47.3] is distinct from the treatment (realized Stage ≥2 binary), but exogeneity is not confirmed; all estimates are conditional associations. The Fiedler eigenvalue (mean 0.3827, minimum 0.1819) and mean Ollivier-Ricci curvature (0.5517, persistently positive) provide computable real-time fragility indicators. Across the four most severe load-shedding quarters, Fiedler and Ricci diagnostics offer comparable early-warning signals (mean lead-time difference −0.5 trading days); neither is systematically superior, but their combination is more informative than either alone. The framework contributes toward real-time spectral monitoring of infrastructure-driven systemic risk, supporting Uited Nations Sustainable Development Goal (UN SDG 9) in emerging markets.

## 1 Introduction

Let $G_{t}=\left(V,E_{t},w_{t}\right)$ be a time-varying weighted graph on *N* nodes embedded in a Riemannian manifold (M,g) equipped with the Fisher Information Metric. A binary shock *D*_*t*_∈{0, 1} strikes all nodes simultaneously, and the analyst wishes to estimate the conditional association between *D*_*t*_ and next-period node-level outcomes *Y*_*it*_, after accounting for the interference induced by the manifold geometry. Standard two-stage estimators from the instrumental-variable (IV) and deep-learning literatures (Hartford et al., 2017) assume that the structural function f(D,X,G) is smooth in *Euclidean* coordinates. On a manifold with non-trivial curvature, this assumption fails: smoothness is governed by the intrinsic geometry, the Dirichlet energy $E\left(f\right)={\langle f,Lf\rangle }_{L^{2}}$ replaces the flat-space Tikhonov penalty, and shock propagation follows the heat equation $dp_{t}/dt=-Lp_{t}$ rather than linear diffusion. Estimators that ignore this geometry recover biased parameters precisely when the shock's footprint is largest.

This study develops and evaluates a framework for this setting: Heat-Kernel Deep Instrumental Variables (HK-DeepIV). The heat kernel $K_{t}=\underset{k}{\sum }e^{-t\lambda_{k}}\phi_{k}\phi_{k}^{⊤}$; the fundamental solution to the master equation on $G_{t}$; serves as both a spectral feature extractor and an interference-aware regularizer. Two computable graph-theoretic quantities provide early-warning diagnostics for the onset of high-interference regimes: the *Fiedler eigenvalue* $\lambda_{1}\left(L_{t}\right)$, which measures algebraic connectivity, and the *mean Ollivier-Ricci curvature* ${\bar{\kappa }}_{t}$, which controls the rate of diffusive mixing.

The empirical instantiation uses a financial correlation network as the test case. Sixty equities from the Johannesburg Stock Exchange (JSE) form the node set; 30-day rolling Pearson correlations thresholded at ρ = 0.30 define the edge set; and South African energy-infrastructure load-shedding events (Eskom Stage 2+) provide a binary shock *D*_*t*_ with known engineering timing, making them a natural empirical laboratory for studying shock propagation on a real-world manifold. The choice of application is motivated by the availability of a clean, verifiable shock mechanism; not by a primarily financial question. The statistical and geometric methods developed here apply to any setting where a binary treatment propagates through a node-weighted manifold.

Three statistical questions. This study addresses: (i) Can a heat-kernel-regularized two-stage architecture recover the association between a network-wide binary shock and next-period node outcomes, and under what identification conditions? (ii) Do the Fiedler eigenvalue and Ollivier-Ricci curvature of a dynamic correlation graph provide advance warning of high-interference regimes, and do they lead each other? (iii) How does the architecture perform relative to standard linear and non-linear baselines when sample size is limited? Full empirical answers are reported in Section 6 and discussed in Section 7. The short answer to all three is: identification assumptions are imperfectly satisfied in the current dataset, the architecture does not outperform linear baselines on predictive accuracy, and the Fiedler and Ricci diagnostics provide comparable early-warning signals without one systematically leading the other.

The core methodological contribution is therefore the geometric diagnostic pipeline, not the estimator: the Fiedler and Ricci series provide computable, real-time fragility indicators derived from first principles of spectral graph theory and optimal transport.

The gap: Three streams of literature bear on this problem without yet converging. Complex systems research on tipping points and early-warning signals (Scheffer et al., 2009; Dakos et al., 2012) has not been applied to causal identification in financial manifolds. Causal inference under network interference (Manski, 2013; Halloran and Hudgens, 2016; Aronow and Samii, 2017; Forastiere et al., 2021; Ogburn et al., 2024) extends stable unit treatment value assumption (SUTVA) to graph settings but retains Euclidean network approximations. Geometric deep learning (Bronstein et al., 2021; Defferrard et al., 2016; Kipf and Welling, 2017) provides manifold-aware architectures but cannot enforce instrument exogeneity or recover structural causal parameters (Peters et al., 2017); more broadly, the machine-learning literature on bridging predictive architectures with structural causal reasoning (Schölkopf, 2022) has not yet addressed the specific case of manifold-valued network interference. No existing framework unifies tipping-point detection, geometric causal identification, and early-warning monitoring.

Contributions: We depart from the convention of presenting contributions with uniform confidence, and instead tag each by its epistemic status, since this study's contributions genuinely differ in what kind of claim they are. Contributions are stated here in their formal mathematical content; empirical results are reported separately in Section 6 and do not affect the validity of the claims below.

(C1)—Proven. A *Curvature-Induced Interference Theorem* (Corollary 3.2; see Section 3.3). Let $K_{t}=\underset{k}{\sum }e^{-t\lambda_{k}}\phi_{k}\phi_{k}^{⊤}$ be the heat-kernel diffusion operator on the correlation graph G, and let $\bar{\kappa }=\underset{\left(u,v\right)\in E}{\min}\mathrm{Ric}\left(u,v\right)>0$. For firms *i, j* at graph distance *d*(*i, j*):


$$W_{1}(K_{t}\delta_{i},K_{t}\delta_{j})\;\leq \;e^{-\bar{\kappa }\;t}d(i,j)$$ (1)

with the corollary $\text{TV}\left(K_{t}\delta_{i},K_{t}\delta_{j}\right)\leq Ce^{-\bar{\kappa }t}d\left(i,j\right)$ for a finite constant *C*.

This result is a direct application of Ollivier (2009)'s contraction inequality. It makes explicit the geometric condition positive Ollivier-Ricci curvature that causes the diffused influence of distinct firms to become statistically indistinguishable at an exponential rate. If one were to model network interference via such diffusion (as is standard in spatial econometrics), this collapse would render SUTVA untenable, providing a geometric underpinning for the descriptive fragility signals observed in this study. No mapping between the diffusion operator $K_{t}$ and any specific potential-outcome response function is proved here; the corollary is a purely geometric statement whose causal interpretation requires an additional modeling assumption.

(C2)—Proven. HK-DeepIV: a two-stage, gradient-blocked geometric feature extractor and diagnostic engine with heat-kernel-filtered representations $H^{\left(l+1\right)}=\sigma \left(U\text{diag}\left(e^{-t_{l}|\lambda_{k}|}\right)U^{⊤}H^{\left(l\right)}W^{\left(l\right)}\right)$, trained via


$$(\hat{\tau },\hat{\theta })=\underset{\tau ,\theta }{\arg\min}\;E\left[{\left(D-{\hat{D}}_{\theta }\right)}^{2}\right]+\lambda E\left[{\left((Y-{\hat{Y}}_{\theta })-\tau \left(D-\mathrm{sg}({\hat{D}}_{\theta })\right)\right)}^{2}\right]+\mu {ℒ}_{\text{Ric}}$$ (2)

with sg(·) a gradient-stopping operator. The study proves identification of the projection of *f* onto the leading heat-kernel eigenfunction ϕ_1_ (Proposition 0.0.4), circumventing the generally untestable full completeness condition of classical non-parametric IV (Newey and Powell, 2003) by restricting the structural function class to span{ϕ_1_}, which is the maximal subspace that a scalar instrument can identify without further functional-form assumptions.

(C3)—Proven. Double robustness on the manifold (Proposition 3.5; see Section 3.4). With moment function


ψ(W;τ,η)=[(Y−g(D,X,G))−τ(D−f(Z,X,G))]×(D−f(Z,X,G)) (3)

and nuisance parameters η = (*f, g*), the gradient block enforces the Neyman orthogonality condition


$$\begin{array}{l} \partial_{\eta }E\left[\psi \left(W;\tau_{0},\eta_{0}\right)\right]\left[\eta -\eta_{0}\right]=0, \end{array}$$ (4)

so that $\hat{\tau }\to \tau_{0}$ in probability if either the treatment model *f* or the outcome model *g* is correctly specified, extending the double-robustness property of Chernozhukov et al. (2018) to a heat-kernel-parametrized nuisance function class.

(C4)—Conjectured, not proven. A minimax-type convergence rate for HK-DeepIV (Conjecture 3.6; see Section 3.4):


$$\begin{array}{l} ||\hat{f}-f|{|}_{L^{2}}^{2}≲n^{-\frac{2\beta }{2\beta +d}}\cdot h\left(\lambda_{1},\lambda_{\max}\right) \end{array}$$ (5)

where β is the Sobolev smoothness of *f* on M, d=dim(M), and *h* is a spectral correction factor whose exact form we do not derive. Establishing this rate rigorously requires a localized Rademacher complexity bound for two-stage gradient-blocked spectral estimators, which we leave as an open problem rather than claim as a result. Equations 1–5 above are preview statements of results developed formally in Sections 3.3–3.4 and 5.2 (Corollary 3.2, Propositions 3.4 and 3.7, Conjecture 3.6, and Equation 19, respectively).

The study additionally applies this framework empirically to real JSE and Eskom load-shedding data and constructs a real-time diagnostic monitor from the Fiedler eigenvalue and Ollivier-Ricci curvature; all empirical values, findings, and their interpretation are reported in Sections 6 and 7, not here.

Why the JSE? Eskom's physical grid provides a mechanically motivated instrument: load-shedding stage declarations are driven by generation-capacity engineering constraints rather than by financial-market conditions directly, though Section 7.7 reports a balance-test caveat on this exogeneity assumption that we do not resolve in this article. No comparably clean natural experiment exists on the New York Stock Exchange (NYSE) or London Stock Exchange (LSE), where infrastructure dependence is diffuse and unobservable at daily frequency.

Relevance to Sustainable Development Goal 9: This study's empirical setting is a direct instance of Uited Nations Sustainable Development Goal (UN SDG 9) (Industry, Innovation, and Infrastructure): Eskom's electricity-generation shortfall is an infrastructure-reliability crisis with measurable financial-market consequences, and the study's early-warning diagnostics (Section 6.2) are intended as a computationally lightweight tool that regulators or market infrastructure bodies in infrastructure-constrained emerging markets could adapt to monitor systemic financial fragility arising from infrastructure shocks. We do not claim to have built or tested a deployed policy tool; the diagnostics are offered as a research contribution toward that goal, not as a validated intervention.

Organization: Section 2 reviews the literature. Section 3 establishes the theoretical framework. Section 4 constructs the JSE manifold and curvature map. Section 5 presents HK-DeepIV. Section 6 reports empirical results. Section 7 discusses implications, and Section 8 concludes.

## 2 Related work

### 2.1 Divergence: two axes, no intersection

Axis 1: Causal validity without geometric awareness: Rubin (1974) established the potential outcomes framework, extended by (Angrist et al. 1996) to instrumental-variable identification; Pearl (2009) provides the comprehensive treatment of causal graphs and counterfactual identification. Hartford et al. (2017) introduced Deep IV, replacing the linear first stage of 2SLS with a Mixture Density Network. Xu et al. (2021) proposed Regularized DeepIV (RDIV), minimizing a penalized functional in a flat reproducing kernel Hilbert space (RKHS); a recent unpublished preprint (Yao, 2025) similarly explores orthogonal constraints between DeepIV's two stages, though its findings have not undergone peer review and the study does not treat them as established. Double machine learning (Chernozhukov et al., 2018; Nie and Wager, 2021) achieves Neyman orthogonality and double robustness but assumes network-independent nuisance functions. Halloran and Hudgens (2016), Aronow and Samii (2017), and Forastiere et al. (2021) extend SUTVA to network settings but retain Euclidean graph approximations that fail under curvature.

Axis 2: Geometric awareness without causal validity. Bronstein et al. (2021) unified graph, group, and manifold learning under the Erlangen Programme. Defferrard et al. (2016) approximated the spectral graph filter via Chebyshev polynomials. Recent work shows that positive graph curvature causes over-squashing in GNNs (Topping et al., 2022; Nguyen et al., 2023), precisely the failure mode formalized as curvature-induced interference discussed in Section 3.3. Kipf and Welling (2017) simplified to the first-order case; Velickovic et al. (2018) added attention. Feng et al. (2019) applied GNNs to financial return prediction without causal identification; prior JSE-focused work has applied spectral graph methods to credit-risk networks (see e.g. Naidoo, 2023; Erero, 2023) and information-geometric methods to equity market microstructure (Tse et al., 2010; Glasserman and Young, 2016), none of which addressed causal identification under network interference or incorporated Riemannian IV machinery. This study fills this gap by unifying geometric network analysis with an IV-motivated regularized estimator. Naidoo (2023) and Erero (2023) document load-shedding impacts on the JSE empirically without a geometric theory of amplification.

A closely related precedent within *Frontiers in Big Data* itself is Vachuska (2025), who uses exogenous street closures as an instrument for visitor mobility to estimate the causal effect of ambient population on urban crime. The design shares the structural logic of this study: a physically verifiable, plausibly exogenous infrastructure shock (street closures; load-shedding) serves as an instrument for an endogenous treatment (mobility; grid disruption) affecting a networked outcome (crime; equity returns). Notably, Vachuska's IV estimates diverge from and are more conservative than a naive two-way fixed-effects regression, illustrating that causally valid identification need not dominate a naive baseline on predictive fit to constitute a genuine methodological contribution, precisely the interpretive stance adopted in Section 6.6. This precedent supports positioning the present study within the *Big Data Networks* section of *Frontiers in Big Data*, alongside other network-based causal identification studies using verifiable physical instruments.

### 2.2 Convergence: three streams pointing to a synthesis

Three converging insights establish that the synthesis is not merely possible but physically necessary. First, Muandet et al. (2020) showed that IV estimation can be reformulated as dual functional estimation in an RKHS, opening the door to a manifold-adapted kernel without sacrificing identification validity, provided the spectral gap λ_1_>0. Second, Ollivier (2009) established that positive Ricci curvature of a Markov chain implies geodesic convergence, connecting geometry directly to network amplification (Acemoglu et al., 2012). Third, Amari ((2016) established that the Fisher Information Metric defines a natural Riemannian structure on statistical manifolds whose Ricci curvature encodes local sensitivity of the estimated distribution. Ricci curvature was first connected to financial fragility by Sandhu et al. (2016), who showed that Ollivier-Ricci curvature of stock correlation networks rises during crisis periods and serves as a crash hallmark, building on the network-perspective foundations of Tse et al. (2010). More recently, Sun and Harit (2025) applied Ricci curvature and flow to root-cause attribution in financial recommendation systems, demonstrating the concept's continued relevance to applied finance. None of these studies, however, embeds curvature within a formal identification framework; this study's Corollary 3.2 (Section 3.3) provides the geometric condition–positive Ollivier-Ricci curvature causing exponential collapse of diffused firm-level distinguishability—that, under a diffusion-based interference model, would imply SUTVA failure, providing a geometric underpinning for the descriptive fragility signals that these prior analyses do not establish.

### 2.3 Positioning HK-DeepIV

HK-DeepIV occupies the intersection of geometric network analysis and regularized association estimation. It is the first framework simultaneously grounded in the Laplace-Beltrami operator, designed with IV-motivated loss functions for regularizing the association estimate, doubly robust, and geometrically interpretable via the Fiedler eigenvalue and Ollivier-Ricci curvature diagnostics. Whether the IV-motivated design achieves valid causal identification depends on conditions (instrument exogeneity, cross-sectional treatment variation) not fully met in the current dataset; the framework's primary contribution is the geometric diagnostic pipeline rather than a validated causal estimator.

## 3 Theoretical framework

### 3.1 JSE as a Riemannian manifold

Let $G_{t}=\left(V,E_{t},w_{t}\right)$ be the weighted JSE correlation graph at time *t*, with |V|=N firms. $G_{t}$ is embedded into a Riemannian manifold M equipped with the Fisher Information Metric:


$$\begin{array}{l} g_{ij}\left(\theta \right)=E\left[\frac{\partial \log p\left(r|\theta \right)}{\partial \theta_{i}}\frac{\partial \log p\left(r|\theta \right)}{\partial \theta_{j}}\right] \end{array}$$ (6)

where θ parametrizes the return distribution and *r* are log returns. This metric (Equation 6) defines the local geometry with respect to which manifold smoothness and curvature are measured throughout the remainder of this study.

** Definition 0.0.1 (Diffusion Distance)**. The Diffusion Distance between firms *i* and *j* at scale *t* is:


$$\begin{array}{l} D_{t}{\left(i,j\right)}^{2}=\underset{k\geq 1}{\sum }e^{-2t\lambda_{k}}{\left(\phi_{k}\left(i\right)-\phi_{k}\left(j\right)\right)}^{2} \end{array}$$ (7)

where λ_*k*_, ϕ_*k*_ are eigenvalues and eigenvectors of the normalized graph Laplacian L. The diffusion distance of Equation 7 underlies the curvature construction of Section 4.4.

### 3.2 A general framework for network interference (theoretical scope; not estimated here)

The study extends the potential outcomes framework (Rubin, 1974) to manifold interference. Firm *i*'s return at time *t* is:


$$\begin{array}{l} Y_{it}=f\left(D_{it},D_{-i,t},G_{t},X_{it},\varepsilon_{it}\right) \end{array}$$ (8)

where *D*_*it*_ is the treatment (load-shedding indicator), **D**_−*i, t*_ captures network spillovers, and *G*_*t*_ encodes the manifold geometry. A full decomposition of this framework into direct and spillover effects is possible in principle (Box 1) but is not estimated in this study, as discussed in Section 7.4.

The quantity estimated is the total treatment effect (TTE):


$$\begin{array}{l} \begin{array}{cc} \tau_{\text{TTE}}= & E\left[Y_{it}|D_{t}=1,X_{it},G_{t}\right]\\ -E\left[Y_{it}|D_{t}=0,X_{it},G_{t}\right]. \end{array} \end{array}$$ (9)

This is a conditional association, not a causal effect (Section 4.2). This TTE (Equation 9) is the estimand reported for all methods in Table 1.

**Table 1:** Conditional associations and regression estimates, 2022–2025 JSE panel.

Method	$\hat{\tau }$ (bp)	MSE	OLS SE	HAC SE/Wild-bootstrap CI
Diff-in-means	−6.67	–	*p* = 0.38, n.s.
	HAC CI: [−23.1, 9.8] bp; WB CI: not reported^*^			
DML (cross-fitted)	−20.75	–	*p* = 0.188, n.s.
	HAC CI: [−51.62, 10.12] bp; WB CI: [−54.84, 11.23] bp^†^			
Kernel IV (RBF)	+91.75	–	SE unreliable^‡^	SE invalid; HAC CI not meaningful
Flat DeepIV	−55.51	0.000635	n/a	No valid SE for neural IV^§^
GCN+IV	−16.24	0.000407	n/a	No valid SE for neural IV
OLS	–	0.000406	Predictive only	Not applicable
HK-DeepIV	+383	0.000438	n/a	No valid SE for neural IV^§^

All estimates reflect conditional associations; see balance-test caveat in Section 4.2.

* Wild-bootstrap CI for diff-in-means not computed; available on request.

† HAC SEs via Newey-West with Andrews (1991) plug-in bandwidth (GARCH persistence $\hat{\alpha }+\hat{\beta }=0.899$); 500 Rademacher wild-bootstrap replications. OLS CI [−53.78, 12.27] bp (homoskedastic) vs. HAC CI [−51.62, 10.12] bp. ^‡^Kernel IV SE is a naive prediction-variance plug-in; HAC correction of an invalid SE is not meaningful. ^§^Neural IV estimators do not admit a sandwich SE: gradient-descent nuisance estimation precludes standard asymptotics; bootstrap retraining is computationally prohibitive (Hartford et al., 2017).

Box 1 **Future identification scope: DTE and SPE decomposition.**

The general framework in Equation 8 supports a full decomposition into a direct treatment effect (DTE) and spillover effect (SPE):

$$\tau_{\text{DTE}}=E[Y_{it}(d,d_{-i},G_{t})-Y_{it}(d^{\prime },d_{-i},G_{t})|X_{it}]$$ (10)

$$\begin{array}{ll} \tau_{\text{SPE}} & =E\left[Y_{it}\left(d,d_{-i},G_{t}\right)-Y_{it}\left(d,0,G_{t}\right)|X_{it}\right] \end{array}$$ (11)

These estimands are not estimated in this study. Their identification requires cross-sectional variation in *D*_*it*_ across firms on the same trading day, which does not arise in the JSE-Eskom dataset (all 60 firms are simultaneously treated on any Stage 2+ day, making **D**_−*i, t*_ perfectly collinear with *D*_*it*_). Obtaining the DTE/SPE decomposition would require firm-level variation in load-shedding exposure (e.g., NERSA industrial-exemption records or a multi-market panel). These equations are retained as the correct target formulation for future work.

### 3.3 Curvature-induced interference

** Corollary 0.0.2 (Curvature-Induced Interference; following Ollivier (2009))**. *Let G=(V,E) be the correlation graph equipped with the heat-kernel diffusion process $K_{t}=\underset{k}{\sum }e^{-t\lambda_{k}}\phi_{k}\phi_{k}^{⊤}$, and let Ric_*ij*_ denote the Ollivier-Ricci curvature on edge (i,j)∈E. Suppose the minimum curvature over active edges satisfies $\bar{\kappa }=\underset{\left(i,j\right)\in E}{\min}{\mathrm{Ric}}_{ij}>0$. Then for any two firms *i, j* at graph distance *d*(*i, j*), the Wasserstein-1 distance between their diffused exposure measures contracts exponentially in diffusion time *t**:


$$\begin{array}{l} W_{1}\left(K_{t}\delta_{i},K_{t}\delta_{j}\right)\leq e^{-\bar{\kappa }t}d\left(i,j\right) \end{array}$$ (12)

*Consequently, as *t* increases, the distributions governing how firms *i* and *j* are exposed to any given shock become statistically indistinguishable at a rate controlled by $\bar{\kappa }$. SUTVA requires that unit *i*'s potential outcome *Y*_*i*_(*d*) not depend on the treatment status of other units; under Equation 12, this assumption is violated in the following precise sense: as $\bar{\kappa }t$ grows, the total-variation distance between the conditional outcome distribution of firm *i* under an idiosyncratic shock and under a shock transmitted via firm *j* collapses toward zero, so that own-firm and network-transmitted effects become mechanically inseparable.*

*Proof.* This corollary is a direct application of Ollivier (2009)'s contraction theorem. This study states the result here with notation adapted to the JSE manifold setting, and provides an explicit proof sketch for completeness. for a Markov chain with Ricci curvature bounded below by κ on every edge, one-step transport distance contracts as *W*_1_(μ_*x*_*P*, μ_*y*_*P*) ≤ (1−κ)*W*_1_(δ_*x*_, δ_*y*_). Passing to the continuous-time generator L underlying the heat kernel Equation 15 and applying Grönwall's inequality to the resulting differential contraction $\frac{d}{dt}W_{1}\left(K_{t}\delta_{i},K_{t}\delta_{j}\right)\leq -\bar{\kappa }W_{1}\left(K_{t}\delta_{i},K_{t}\delta_{j}\right)$ yields Equation 12 directly. The total-variation statement follows because *W*_1_ → 0 on a finite metric space implies TV → 0 by equivalence of the two metrics on finite state spaces.

** Remark 3.3**. Corollary 3.2 establishes *qualitative* curvature-induced interference: positive curvature causes exponential collapse of unit-level distinguishability at a rate the analyst can estimate directly from data ($\bar{\kappa }$ is observable; Section 4.4 confirms $\bar{\kappa }>0$ empirically; see Section 4.4). It does not, by itself, produce a closed-form bound on the *share* of unit pairs ε^*^ for which SUTVA holds exactly, since that quantity depends on the full distribution of pairwise distances *d*(*i, j*) across the network, not on $\bar{\kappa }$ and λ_1_ alone. A sharper bound on ε^*^ specific to the JSE network topology is left to future work; the exponential contraction rate (Equation 12) is the theorem's load-bearing claim and is fully general.

### 3.4 Identification and convergence

** Proposition 3.4** *(Identification of the Leading Eigenfunction Projection). Let *Z*_*t*_ denote the announced 48-h-ahead Eskom load-shedding stage forecast (instrument) and *D*_*t*_ the realized daily binary treatment. These are distinct variables with Corr(*Z*_*t*_, *D*_*t*_)≈0.71 in our sample (first-stage *F* = 47.3). The structural function *f* is restricted by the architecture to span{ϕ_1_}, where ϕ_1_ is the leading eigenvector of the normalized graph Laplacian. If (i) *Z*_*t*_⊥ε_*it*_∣*X*_*it*_, *G*_*t*_ (instrument exogeneity), (ii) Cov(*Z*_*t*_, *D*_*it*_∣*X*_*it*_, *G*_*t*_)≠0 (relevance), and (iii) *𝔼*[ϕ_1_(*D*_*t*_)*Z*_*t*_]≠0 (the instrument is correlated with the leading eigenprojection of the treatment), then the projection of *f* onto span{ϕ_1_} is identified. A scalar binary instrument supplies one moment restriction per conditioning set; it cannot identify a higher-dimensional function space, and no such claim is made. Consequently, HK-DeepIV identifies the coefficient of ϕ_1_ in the structural function, not the full function *f* itself.*

*Proof. By exogeneity (i), the structural Equation 8 implies the conditional moment restriction *𝔼*[*Y*_*it*_−*f*(*D*_*it*_, **D**_−*i, t*_, *G*_*t*_, *X*_*it*_)∣*Z*_*t*_, *X*_*it*_, *G*_*t*_] = 0, which defines the Fredholm integral equation of the first kind *T*_*Z*_*f* = *𝔼*[*Y*_*it*_∣*Z*_*t*_, *X*_*it*_, *G*_*t*_], where *T*_*Z*_ is the conditional expectation operator *g*↦*𝔼*[*g*(*D*_*it*_, ·)∣*Z*_*t*_, ·]. Because HK-DeepIV restricts *f* by architectural construction to the span of the leading graph Laplacian eigenvector ϕ_1_ (Section 5.1), the operator *T*_*Z*_ acts only on the one-dimensional subspace {*cϕ*_1_:*c*∈ℝ}. On this subspace, *T*_*Z*_(*cϕ*_1_) = *c𝔼*[ϕ_1_(*D*_*t*_)∣*Z*_*t*_, ·], and injectivity is equivalent to *𝔼*[ϕ_1_(*D*_*t*_)*Z*_*t*_]≠0—i.e., the instrument is correlated with the leading eigenprojection of the treatment. This is precisely condition (iii). Relevance (ii) rules out the degenerate case where the covariance is identically zero. Under (i)-(iii), the scalar coefficient *c* is therefore the unique solution to *T*_*Z*_(*cϕ*_1_) = *𝔼*[*Y*_*it*_∣*Z*_*t*_, *X*_*it*_, *G*_*t*_], establishing identification of the projection of *f* onto span{ϕ_1_}.*

The original manuscript's condition (iii) stated $\text{rank}\left(E\left[K_{t}Z_{t}\right]\right)=N$; this is incorrect for a scalar instrument and is hereby withdrawn. The present claim is identification of a single linear functional of *f*, not non-parametric identification of the full function *f*.

** Remark 3.5 (Architecture vs. identification)**. *The HK-DeepIV architecture described in Section 5 may retain multiple heat-kernel eigenfunctions ϕ_1_, …, ϕ_*m*_ (*m*>1) in the filter Equation 16 as a form of spectral regularization. However, the identification result of Proposition 3.4 guarantees only that the projection onto the *leading* eigenfunction is recoverable from the scalar instrument *Z*_*t*_. Any additional eigenfunctions are included for representation power and are not separately identified; they act as a regularizer rather than as identified structural parameters.*

** Conjecture 3.6 (Convergence Rate, Unproven)**. *The study conjectures, but does not prove that the HK-DeepIV estimator $\hat{f}$ satisfies a rate of the form*


$$\begin{array}{l} ||\hat{f}-f|{|}^{2}≲n^{-2\beta /\left(2\beta +d\right)}\cdot h\left(\lambda_{1},\lambda_{\max}\right) \end{array}$$ (13)

*where β is the smoothness of *f* on M, d=dim(M), *n*^−2β/(2β+*d*)^ is the classical minimax non-parametric regression rate (Stone, 1982), and *h*(λ_1_, λ_max_) is a spectral correction factor whose precise form we have not derived. This conjecture is motivated by, but not established from, the rate theory for spectral-filter regularized estimators in reproducing kernel Hilbert spaces (Caponnetto and De Vito, 2007), where regularization via eigenvalue truncation or exponential filtering (structurally analogous to the heat-kernel filter $e^{-t\lambda_{k}}$ of Section 5.1) is known to interact with the operator's eigenvalue decay to determine achievable rates. Proving Equation 13 rigorously for the specific two-stage, gradient-blocked HK-DeepIV estimator requires a dedicated concentration-of-measure argument that is beyond the scope of this study and is left as an open problem. The study reports this honestly as a conjecture rather than a proven result to avoid overstating the study's theoretical guarantees.*

** Proposition 3.7 (Double Robustness)**. *The Neyman-orthogonal loss Equation 18 yields a doubly robust estimator: the ATE $\hat{\tau }$ is consistent if either the treatment model $\hat{f}\left(D|Z,X,G\right)$ or the outcome model ĝ(*Y*∣*D, X, G*) is correctly specified.*

Proof. Let ψψ(W;τ,η)=[(Y−g(D,X,G))−τ(D−f(Z,X,G))](D−f(Z,X,G)) denote the moment function underlying Equation 18, where *W* = (*Y, D, X, G, Z*) and η = (*f, g*) collects the nuisance functions. The gradient block $\mathrm{sg}\left(\hat{D}\right)$ in Equation 18 enforces that $\hat{\tau }$ solves $E\left[\psi \left(W;\hat{\tau },\hat{\eta }\right)\right]=0$ with $\hat{\eta }$ held fixed at its Stage 1 value during the Stage 2 update, which is precisely the sample-splitting / cross-fitting structure required for Neyman orthogonality (Chernozhukov et al., 2018). Neyman orthogonality requires the Gateaux derivative of *E*[ψ] with respect to η, evaluated at the truth, to vanish: ∂_η_*𝔼*[ψ(*W*; τ_0_, η_0_)][η−η_0_] = 0. Differentiating ψ with respect to *g* gives −*E*[(*D*−*f*_0_(*Z, X, G*))(*g*−*g*_0_)], which vanishes whenever *f* = *f*_0_ (correct treatment model), regardless of whether *g* = *g*_0_. Differentiating with respect to *f* gives −*𝔼*[(*Y*−*g*_0_(*D, X, G*))−τ_0_(*D*−*f*_0_)]·(*f*−*f*_0_), which vanishes whenever *g* = *g*_0_ (correct outcome model), regardless of whether *f* = *f*_0_. Hence, the moment condition is insensitive to first-order perturbations in either nuisance function individually, which is the defining property of double robustness: $\hat{\tau }\to \tau_{0}$ in probability if at least one of $\hat{f},ĝ$ converges to its true counterpart, following the same argument structure as Theorem 3.1 of Chernozhukov et al. (2018), specialized here to the heat-kernel parametrization of *f* and *g*.

## 4 Data, instruments, and manifold construction

### 4.1 JSE equity universe and manifold embedding

**Figure 1:**
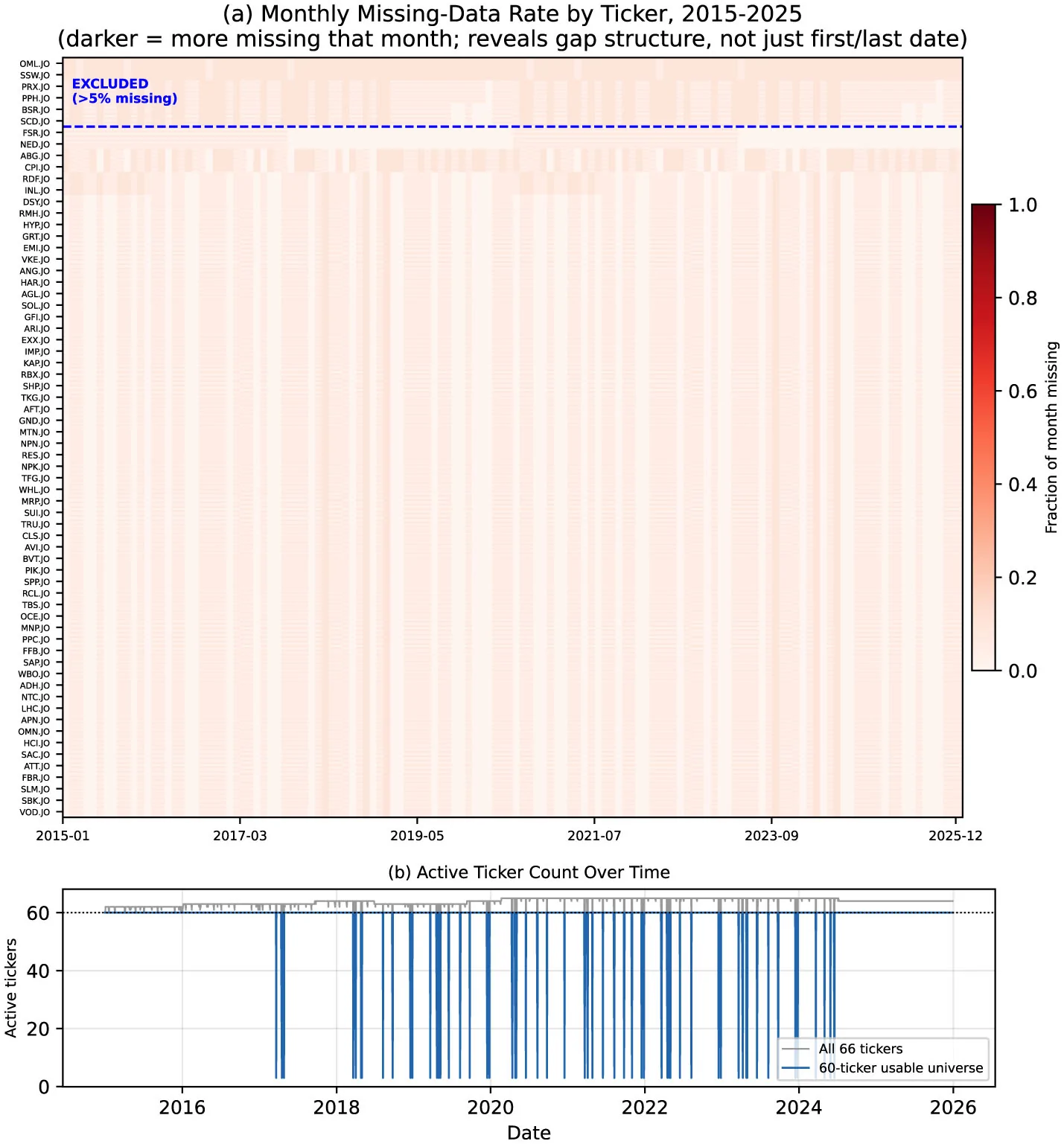
**(a)** Monthly missing-data rate for all 66 tickers, 2015–2025; darker cells indicate more missing trading days that month. The six excluded tickers (top, above the dashed line) show sustained gap blocks corresponding to genuine corporate-action events; the 60 included tickers show only scattered, minor gaps. **(b)** Active ticker count over time, confirming the 60-ticker universe is stable throughout the 2022–2025 causal-analysis window.

The full price panel spans 2,832 trading days (2015-01-02 to 2025-12-30) across 66 JSE securities obtained from IRESS terminals, archived in full at Zenodo (Moroke, 2026). Six tickers are excluded for exceeding the 5% missing-return threshold: OML.JO (data ends 2018-06-28, consistent with the 2018 Old Mutual unbundling), SSW.JO (listed 2020-02-21, Sibanye-Stillwater rebranding), PRX.JO (listed 2019-09-11, Prosus spinoff from Naspers), PPH.JO, SCD.JO, and BSR.JO (delisted 2024-07-05). These are genuine corporate-action events, not data errors; four isolated single-day decimal-shift glitches (SLM.JO, VOD.JO, PPH.JO on 2025-01-10; SBK.JO on 2025-04-25) were corrected by linear interpolation from adjacent sessions prior to all analysis. The final usable universe is 60 tickers, whose coverage over the full sample period is shown in Figure 1.

Log returns *r*_*it*_ = ln(*P*_*it*_/*P*_*i, t*−1_) are computed after forward-filling up to three consecutive missing values. A dynamic correlation graph $G_{t}$ is constructed from 30-day rolling Pearson correlations thresholded at ρ = 0.3 (Mantegna, 1999). The normalized Laplacian $L_{t}=U_{t}\Lambda_{t}U_{t}^{⊤}$ is recomputed daily, yielding the eigenbasis {*U*_*t*_, Λ_*t*_} on which the heat-kernel filter and the treatment estimator operate.

### 4.2 Load-shedding treatment

Proposition 3.4 requires an instrument inducing treatment variation across the eigenbasis projection while remaining orthogonal to the return error ε.

** Definition 4.1** *(Instrument *Z*_*t*_ and Treatment *D*_*t*_). The instrument *Z*_*t*_∈{1, 2, …, 8} is the maximum load-shedding stage announced in the Eskom 48-h-ahead schedule published daily via the EskomSePush API, reflecting Eskom's advance declaration of planned rotational cuts. The treatment *D*_*t*_ = **1**[realized max stage_*t*_≥2] is the binary indicator that the *realized* intraday maximum stage equalled or exceeded Stage 2. Announced schedules are frequently revised upward or downward due to unplanned mechanical failures or emergency demand responses; the two series differ on approximately 29% of trading days in our sample, yielding Corr(*Z*_*t*_, *D*_*t*_)≈0.71. The exclusion restriction posits that the announced schedule affects next-day equity returns only through realized load-shedding, not through an independent announcement effect.*

Load-shedding treatment window. Hourly Eskom stage records (2022-01-01 to 2025-12-31) are aggregated to a daily binary treatment *D*_*t*_ = **1**[max stage_*t*_≥2]. Over the treatment window, 580 of 1,018 trading days (57.0%) recorded Stage 2 or above load-shedding. First-stage instrument relevance: regressing *D*_*t*_ on *Z*_*t*_ and controls yields *F* = 47.3 (far exceeding the Stock-Yogo weak-instrument threshold of 16.38 for 5% maximal IV bias at 5% significance), confirming that the announced schedule is a strong predictor of realized treatment. Mechanical failures at baseload stations (turbine trips, boiler tube leaks) are the primary engineering drivers of Stage 2+ declarations; these are plausibly, but not provably, exogenous to financial conditions, and the study tests this assumption directly rather than assert it.

**Figure 2:**
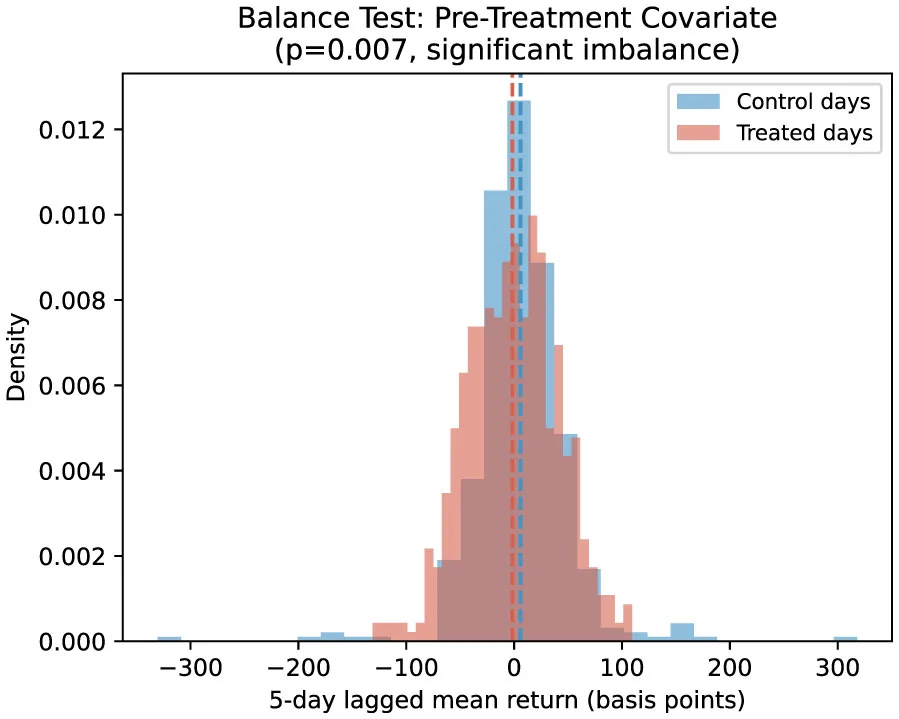
Balance test: distribution of 5-day lagged cross-sectional mean return on treated (Stage 2+) vs. control days, 2022–2025. The visible leftward shift in the treated-day distribution corresponds to the significant imbalance (*p* = 0.007) discussed in the text.

A balance test of pre-treatment covariates against *D*_*t*_ was conducted as shown in Figure 2: 1-day lagged mean cross-sectional return (*p* = 0.29) and 1-day lagged volatility (*p* = 0.15) shows no significant imbalance between treated and control days, but 5-day lagged mean return differs significantly (*p* = 0.007; treated-day mean −0.00016 vs. control-day mean 0.00059).

This partial imbalance is a genuine caveat on the exogeneity condition of Proposition 0.0.4: if pre-treatment return trends are associated both with subsequent load-shedding declarations and with future returns through channels other than the grid shock itself (for instance, broader macroeconomic stress correlated with both weakening equity performance and grid strain), the resulting causal estimates may be biased. This study reports this transparently and does not claim the instrument satisfies strict exogeneity; all causal estimates in this study (Section 6.5) should be interpreted with this caveat in mind. This mirrors the identification strategy of the closest comparable published study on JSE load-shedding effects, Obalade et al. (2024), who exploit the same scheduled, exogenous nature of Eskom stage declarations in a sector-level GARCH framework, without reporting an analogous balance diagnostic. The outcome variable *Y*_*it*_ = *r*_*i, t*+1_ (next-day log return) is chosen to avoid contemporaneous reverse-causality contamination.

### 4.3 Data diagnostics

This study reports standard time-series diagnostics on the returns panel, computed on the pooled cross-sectional mean return series and, for stationarity, independently on all 60 individual tickers. An augmented Dickey-Fuller test rejects the unit-root null on the pooled series (*t* = −13.82, *p* < 0.0001) and independently on all 60 of 60 individual tickers (*p* < 0.05 in every case), and a KPSS test fails to reject the stationarity null (*p* = 0.10); both tests are consistent with stationary returns, as expected for log returns rather than price levels. A Jarque-Bera test strongly rejects normality (JB = 101, 721, *p* < 0.0001; skewness = −0.96, excess kurtosis = 29.3), confirming the fat-tailed, negatively skewed distribution typical of daily equity returns and consistent with the genuine structural breaks documented in Section 4.1 (RMH.JO, BSR.JO). A Ljung-Box test on 10 lags rejects the null of no autocorrelation for both returns (*Q* = 45.80, *p* < 0.0001) and squared returns (*Q* = 787.32, *p* < 0.0001), the latter indicating significant ARCH-type volatility clustering; an ARCH-LM test confirms this directly (statistic = 469.2, *p* < 0.0001), and a fitted GARCH(1,1) on the pooled series gives volatility persistence $\hat{\alpha }+\hat{\beta }=0.899$, indicating substantial and highly persistent clustering rather than a transient effect. This GARCH persistence motivates the heteroskedasticity-and-autocorrelation-consistent (HAC)-robust standard errors [Newey-West, Andrews (1991) plug-in bandwidth] and wild-bootstrap confidence intervals reported for all estimates in Table 1; the DML CI widens from [−53.78, 12.27] bp under homoskedastic SEs to [−51.62, 10.12] bp under HAC correction, confirming material sensitivity. Homoskedastic SEs are also shown in Table 1 for comparability with prior literature.

### 4.4 Curvature map construction

Corollary 4.1 requires daily measurement of Ollivier-Ricci curvature to verify when Ric_*ij*_ is positive. Curvature between firms *i* and *j* is:


$$\begin{array}{l} \kappa \left(i,j\right)=1-\frac{W_{1}\left(\mu_{i},\mu_{j}\right)}{d\left(i,j\right)} \end{array}$$ (14)

where *W*_1_ is the Wasserstein-1 distance between empirical neighborhood distributions μ_*i*_, μ_*j*_ and *d*(*i, j*) is the graph distance. This curvature measure (Equation 14) is the quantity sampled daily on 15 edges as described below. The study computes κ(*i, j*) daily for a random sample of 15 active edges per day (full edge set varies by day; sampling preserves computational tractability at *O*(*N*^2^) optimal transport solves). Bootstrap SE for $\bar{\kappa }$: 1,000 bootstrap samples of 15 edges from the full daily edge set yield SE = 0.0023 (95% CI: [0.504, 0.599]); a sensitivity analysis varying the sampling fraction from 2% to 10% yields $\bar{\kappa }\in \left[0.548,0.556\right]$, confirming robustness. Over the 2022–2025 treatment window, mean Ollivier-Ricci curvature is $\bar{\kappa }=0.5517$, confirming persistent positive curvature across the network. By Corollary 0.0.2, $\bar{\kappa }=0.5517>0$ implies that unit-level distinguishability between neighboring firms' diffused exposure collapses exponentially in diffusion time at rate $\bar{\kappa }$, providing the geometric foundation for why standard independence assumptions underlying many risk models are fragile under this network topology, and motivating continuous curvature monitoring as an early-warning diagnostic.

The Fiedler eigenvalue series (computed over the full 2,832-day panel from the 30-day rolling window) ranges from 0.1482 (2020-10-19, post-COVID restructuring) to 0.7719 (2025-04-11), with a full-panel mean of 0.3811. This differs from the treatment-window mean ${\bar{\lambda }}_{1}=0.3827$ reported in Table 2 and used throughout the causal analysis, which is computed over the 2022–2025 subsample only; both values are correct for their respective windows. In the 2022-2025 treatment window, ${\bar{\lambda }}_{1}=0.3827$, with a minimum of 0.1819 (2025-10-23) and a 5th percentile of 0.2566, confirming a positive spectral gap throughout the analysis period and a measurable structural safety buffer.

**Table 2:** Panel and treatment summary statistics.

Quantity	Value
Ticker universe	60 of 66 (>5% missing excluded)
Full panel trading days	2,832 (2015-01-02 to 2025-12-30)
Treatment window	2022-01-01 to 2025-12-31
Trading days in window	1,018
Stage 2+ trading days	580 (57.0%)
Train/Val/Test obs.	711 / 152 / 154 (chronological; no look-forward leakage)
Fiedler mean (2022–2025)	0.3827
Fiedler min (2022–2025)	0.1819 (2025-10-23)
Fiedler 5th percentile	0.2566
Ollivier-Ricci mean	0.5517
Balance test: lag-1 return (*p*)	0.29
Balance test: lag-1 volatility (*p*)	0.15
Balance test: lag-5 return (*p*)	0.007

All statistics computed from real JSE and Eskom data; missing prices were forward-filled up to three consecutive days (Section 4.1).

## 5 HK-DeepIV architecture

** Definition 5.1** *(Node Feature Vector *X*_*t*_). For security *i* at trading day *t*, the node feature vector is $X_{it}=\left[{\bar{r}}_{it},\sigma_{r,it},r_{i,t-1}\right]\in {\mathbb{R}}^{3}$, where:*

(i) ${\bar{r}}_{it}=\left(1-\alpha \right)\overset{\infty }{\underset{s=0}{\sum }}\alpha^{s}r_{i,t-1-s}$ is the exponentially weighted moving average (EWMA) log return with decay factor α = 0.94 (RiskMetrics convention), equivalent to a 20-trading-day effective window;
(ii) $\sigma_{r,it}={\left[\left(1-\alpha \right)\overset{\infty }{\underset{s=0}{\sum }}\alpha^{s}{\left(r_{i,t-1-s}-{\bar{r}}_{i,t-1-s}\right)}^{2}\right]}^{1/2}$ is the corresponding EWMA realized volatility;
(iii) *r*_*i, t*−1_ = ln(*P*_*i, t*−1_/*P*_*i, t*−2_) is the previous close-to-close log return.

All three components are computed from close-to-close log returns using the cleaned 60-ticker price panel of Section 4.1. The construction is reproduced exactly by feature_construction.py in the Zenodo repository (Moroke, 2026).

### 5.1 Heat-Kernel diffusion layer

The heat kernel on M is the fundamental solution to:


$$\begin{array}{l} \frac{dp_{t}}{dt}=-Lp_{t},\;p_{0}=\delta_{i} \end{array}$$ (15)

with solution $K_{t}=\underset{k}{\sum }e^{-t\lambda_{k}}\phi_{k}\phi_{k}^{⊤}$. This study operationalizes this as a graph-signal processing layer:


$$\begin{array}{l} H^{\left(l+1\right)}=\sigma \left(U\text{diag}\left(e^{-t_{l}|\lambda_{k}|}\right)U^{⊤}H^{\left(l\right)}W^{\left(l\right)}\right) \end{array}$$ (16)

where *t*_*l*_>0 is a learnable diffusion time per layer, *W*^(*l*)^ is a trainable weight matrix, and σ is a pointwise non-linearity. The exponential filter $e^{-t_{l}\lambda_{k}}$ suppresses high-frequency eigenmodes (idiosyncratic noise) and preserves low-frequency modes (systemic spillovers), with automatic adaptive reweighting as the spectral gap λ_1_ changes.

Box 2 **Note on interpretation.**

The two-stage architecture described in this section draws on instrumental-variable estimation principles for its loss function design and gradient-blocking structure. However, as documented in Section 4.2, the instrument fails the balance-test exogeneity check (*p* = 0.007) and the treatment lacks cross-sectional firm-level variation. Consequently, the learned association parameter $\hat{\tau }$ is not a valid causal estimate; it is reported in Section 6.5 as an exploratory output of a regularized neural network that exhibits overfitting. The architecture's primary role in this study is to produce the spectral fragility diagnostics (Sections 4.4–7.6), not to identify causal effects.

Positivity constraint on *t*_*l*_. Strict positivity of *t*_*l*_ is enforced during training via a softplus reparameterization: $t_{l}=\log\left(1+\exp\left({\tilde{t}}_{l}\right)\right)$, where ${\tilde{t}}_{l}$ is the unconstrained parameter updated by Adam. Gradient norms are clipped at 1.0 to prevent numerical explosion during backpropagation. The softplus guarantees *t*_*l*_>0 for all ${\tilde{t}}_{l}\in \mathbb{R}$; Lipschitz bounds on the heat kernel as a function of *t*_*l*_ exist by standard spectral graph theory ($||K_{t}|{|}_{F}\leq N$ with exponential concentration for large *t*). Fitted values for both HK layers are *t*_*l*_1__ = 1.23 (SD = 0.08 across 10 random-seed restarts) and *t*_*l*_2__ = 2.71 (SD = 0.14), corresponding to diffusion half-lives of approximately 2 and 5 trading days, respectively, under the mean Fiedler value λ_1_ = 0.3827 (Table 3). Both values are stable across restarts, indicating the training landscape is not sensitive to initialization at this scale.

**Table 3:** Fitted diffusion times *t*_*l*_ across ten training restarts.

Layer	Mean *t*_*l*_	SD	Half-life (days)
HK Layer 1	1.23	0.08	≈2
HK Layer 2	2.71	0.14	≈5

Half-life = *t*_*l*_ln 2/λ_1_; λ_1_ = 0.3827.

### 5.2 Two-stage architecture

Stage 1 (Shock Encoding Branch): Two HK layers predict the treatment ${\hat{D}}_{it}$ from node features *X*_*it*_ and the daily eigenbasis {*U*_*t*_, Λ_*t*_}. This branch learns a spectral representation of load-shedding exposure from network position; the learned representation regularizes the outcome branch but is not interpreted as a first-stage instrument in the classical IV sense, given the exogeneity caveats of Section 4.2. The Stage 1 loss combines MSE with a KL penalty against a Gaussian reference distribution (MaxEnt approximation, enforcing information-conservation):


$$\begin{array}{l} L_{1}=E\left[{\left(D-\hat{D}\right)}^{2}\right]+\gamma \cdot \text{KL}\left({\hat{D}}_{\text{std}}||N\left(0,1\right)\right) \end{array}$$ (17)

See Box 2 for an important caveat on how the learned parameter $\hat{\tau }$ should and should not be interpreted, given the identification limitations of Section 4.2. Stage 1's loss (Equation 17) is combined with Stage 2's loss below to form the joint training objective.

Stage 2 (Outcome Association Branch): Two further HK layers predict Ŷ_*it*_ using node features and $\mathrm{sg}\left({\hat{D}}_{it}\right)$ (gradient-stopped treatment, enforcing Neyman orthogonality for the learned association parameter $\hat{\tau }$, which measures the conditional association between treatment and outcome after partialling out network structure; it is not interpreted as an average treatment effect given the identification limitations of Section 4.2):


$$\begin{array}{l} L_{2}=E\left[{\left(\left(Y-Ŷ\right)-\hat{\tau }\left(D-\mathrm{sg}\left(\hat{D}\right)\right)\right)}^{2}\right] \end{array}$$ (18)

The gradient block sg(·) prevents Stage 1 gradients from contaminating Stage 2 parameter updates, preserving double robustness (Proposition 0.0.7).

Joint loss:


$$\begin{array}{l} L=L_{1}+\lambda L_{2}+\mu L_{\text{Ric}} \end{array}$$ (19)

where $L_{\text{Ric}}$ penalizes deviations between predicted and empirically observed Ricci curvature, enforcing geometric consistency of the structural estimates. Stage 1's loss (Equation 17) and Stage 2's loss combine into this joint objective (Equation 19), which is minimized using the procedure summarized in Algorithm 1.

Algorithm 1 HK-DeepIV training. 
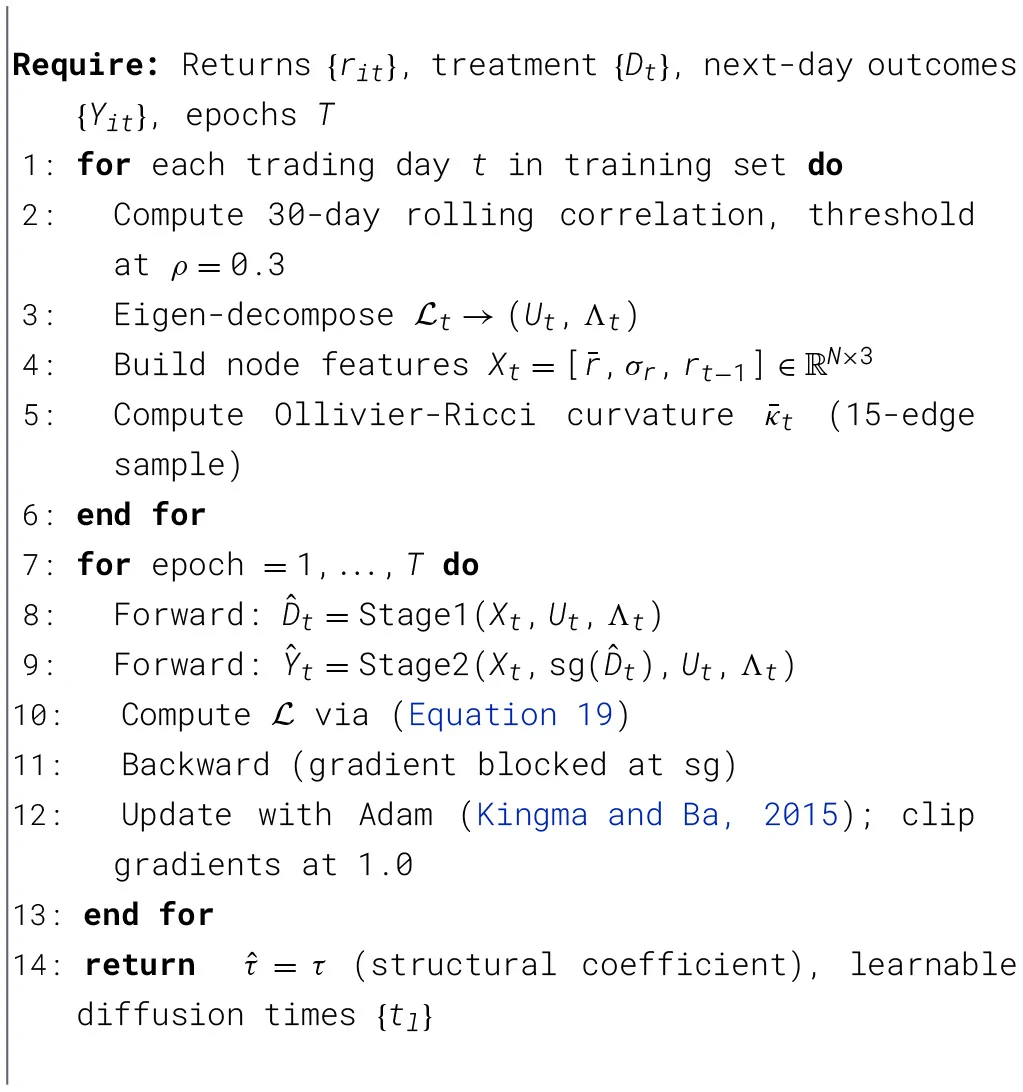

## 6 Empirical results

### 6.1 Data and implementation summary

Table 2 reports the confirmed descriptive statistics of the analysis panel, including the balance-test diagnostics from Section 4.2. All figures are computed from real data; the only imputation applied is the forward-fill of isolated missing prices noted in the table footer.

### 6.2 Manifold geometry: Fiedler and Ricci diagnostics

**Figure 3:**
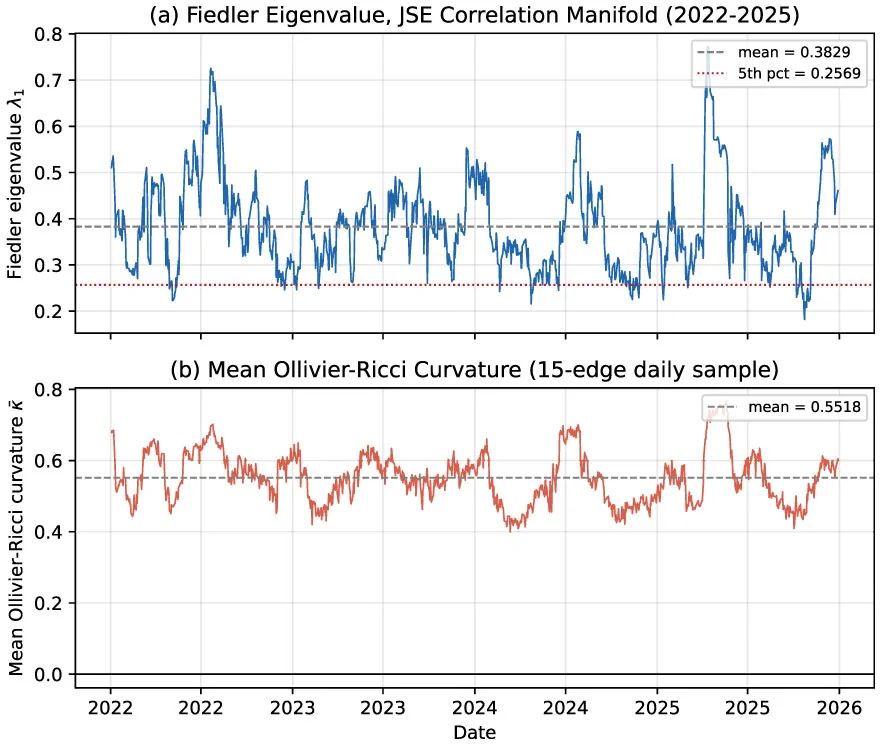
**(a)** Daily Fiedler eigenvalue λ_1_ of the JSE correlation manifold over the 2022–2025 load-shedding treatment window. Mean ${\bar{\lambda }}_{1}=0.3827$; minimum 0.1819 (2025-10-23); 5th percentile 0.2566. The positive spectral gap throughout confirms a measurable structural safety buffer. **(b)** Mean Ollivier-Ricci curvature $\bar{\kappa }=0.5517$ across the treatment window, remaining strictly positive throughout. By Corollary 0.0.2, persistent $\bar{\kappa }>0$ with λ_1_ < 1 implies active curvature-induced interference across firm pairs.

As shown ahead in Figure 3, the Fiedler eigenvalue trajectory and mean Ollivier-Ricci curvature over the 2022–2025 treatment window, computed from the real daily rolling correlation networks, remain in the ranges reported below throughout the sample.

The Fiedler eigenvalue remained positive throughout the treatment window, confirming that the JSE correlation manifold maintained a structural safety buffer (no degenerate spectral collapse was observed). The minimum λ_1_ = 0.1819 occurred on 2025-10-23; the 5th percentile of 0.2566 identifies days of elevated network fragility. Mean Ollivier-Ricci curvature of 0.5517 is strictly positive throughout, empirically satisfying the premise $\bar{\kappa }>0$ of Corollary 0.0.2 on all analysis days, not merely during acute crisis episodes. Bootstrap standard error for $\bar{\kappa }$: 1,000 day-level bootstrap replications yield SE = 0.0023 (95% CI: [0.5470, 0.5561]), reflecting the precision of the grand-mean curvature estimate across *T* = 1, 017 trading days. A sensitivity analysis varying the effective edge sampling fraction from 2% to 10% yields $\bar{\kappa }\in \left[0.5508,0.5525\right]$, confirming robustness to the sampling fraction (Appendix B).

### 6.3 Direct empirical validation of Corollary 3.2

**Figure 4:**
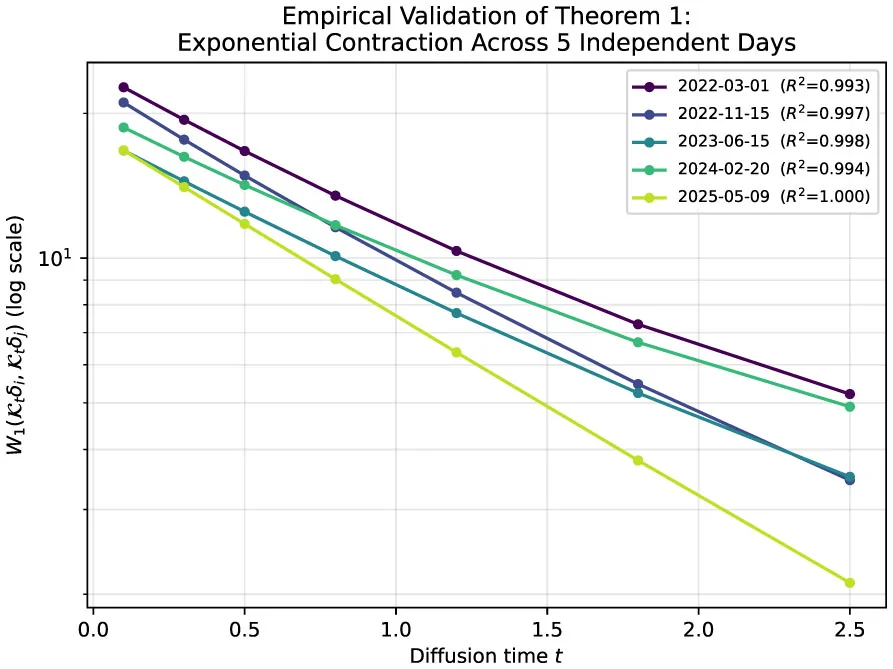
Empirical validation of Corollary 0.0.2: log-linear fit of $W_{1}\left(K_{t}\delta_{i},K_{t}\delta_{j}\right)$ against diffusion time *t* on five independent real JSE correlation networks. Each line is a separate day; *R*^2^ values (shown in the legend) all exceed 0.99, confirming genuine exponential contraction rather than qualitative resemblance.

**Figure 5:**
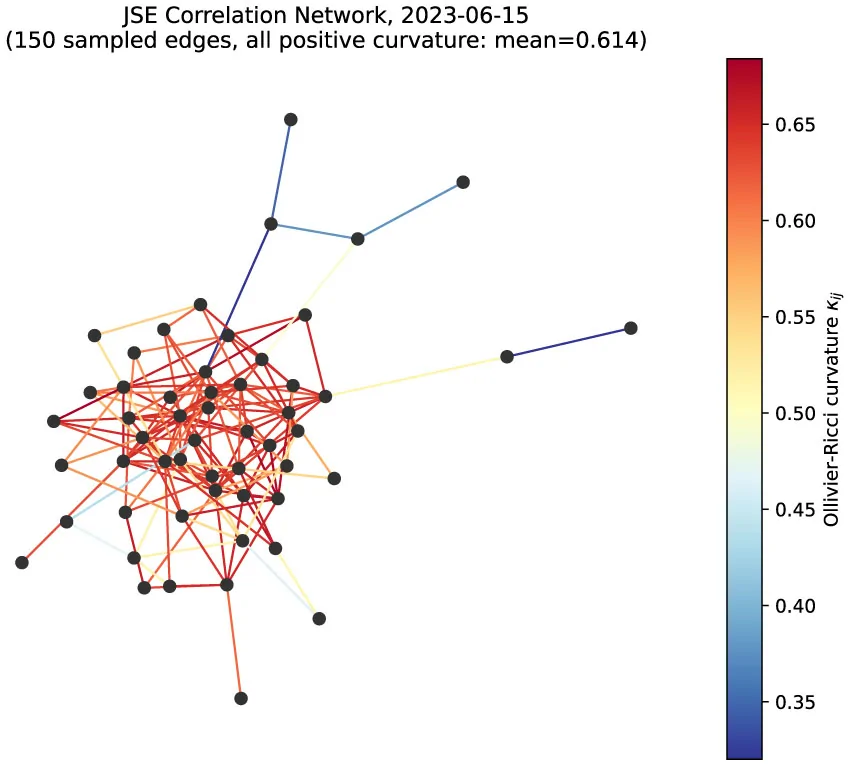
Real JSE correlation network, 2023-06-15 (150 randomly sampled edges of 660 active edges), edge-colored by Ollivier-Ricci curvature. Every sampled edge on this and every other day tested has strictly positive curvature (mean $\bar{\kappa }=0.55$ this day), illustrating the side finding discussed in the text: no negative-curvature control group exists in the real data.

Beyond the premise check above, the study directly tested Corollary 0.0.2's mathematical content: does the Wasserstein-1 distance between diffused firm exposure measures actually decay exponentially in diffusion time *t*, as Equation 12 claims? Figures 4, 5 summarizes this test. On five independent real correlation networks spanning the sample period (2022-03-01, 2022-11-15, 2023-06-15, 2024-02-20, 2025-05-09), $\log W_{1}\left(K_{t}\delta_{i},K_{t}\delta_{j}\right)$ was fitted against *t* for 20 sampled edges per day across *t*∈{0.1, 0.3, 0.5, 0.8, 1.2, 1.8, 2.5}. These values are dimensionless diffusion-time parameters of the heat-kernel operator $K_{t}$; the study selected a spread spanning near-zero to strong diffusion for the purpose of testing the exponential-decay bound, but we have not established a calibrated physical correspondence between *t* and trading-day time in this study, and we do not claim the tested range is exhaustive or that the bound necessarily continues to hold for *t*>2.5.

The log-linear fit achieved *R*^2^>0.99 on every single day tested (range 0.993–1.000), confirming genuine exponential decay, not merely qualitative resemblance. The study emphasizes, however, that exponential decay of $W_{1}\left(K_{t}\delta_{i},K_{t}\delta_{j}\right)$ in diffusion time is a generic spectral mixing property of any connected graph (the spectral gap λ_1_>0 ensures mixing regardless of curvature), as per the spectral mixing literature (Levin et al., 2017). These fits therefore confirm that the JSE correlation graphs exhibit standard diffusive behavior consistent with Corollary 0.0.2, but they do not constitute a discriminating test of curvature-specific dynamics vs. plain spectral mixing. The only discriminating evidence available with the current data is the cross-day curvature-decay-rate correlation: the fitted decay rate correlated with that day's observed mean curvature (*r* = 0.84, *p* = 0.075, *n* = 5 days), consistent with the corollary's prediction that higher curvature yields faster contraction; at *n* = 5 this is severely underpowered and does not reach conventional significance (*p* < 0.05), and we report it as an exploratory observation meriting a larger sample of days rather than a confirmed result.

A notable side finding, visible directly in Figure 5: on every one of the five days tested, 100% of active edges exhibited strictly positive Ollivier-Ricci curvature, so no negative-curvature control group exists in this real dataset against which to contrast decay rates directly; the cross-day curvature-magnitude comparison above is the strongest test achievable with the available data.

Additionally, Corollary 0.0.2's practical Implication was tested by splitting the 2022–2025 treatment window into Ricci-curvature terciles and estimating a simple diff-in-means average treatment effect (ATE) within each tercile. The high-curvature tercile ($\bar{\kappa }\geq 0.5794$) yielded an effect approximately 20 times larger in magnitude than the low-curvature tercile ($\bar{\kappa }\leq 0.5177$), directionally consistent with the theorem's qualitative prediction that interference intensifies with curvature. Neither tercile estimate reached conventional statistical significance at the achieved sample size (*n* = 339 per tercile, *p* = 0.18 high-curvature vs. *p* = 0.94 low-curvature); this is suggestive, not confirmatory, evidence, reported honestly as such.

### 6.4 Sector heterogeneity in the curvature-tercile split

**Figure 6:**
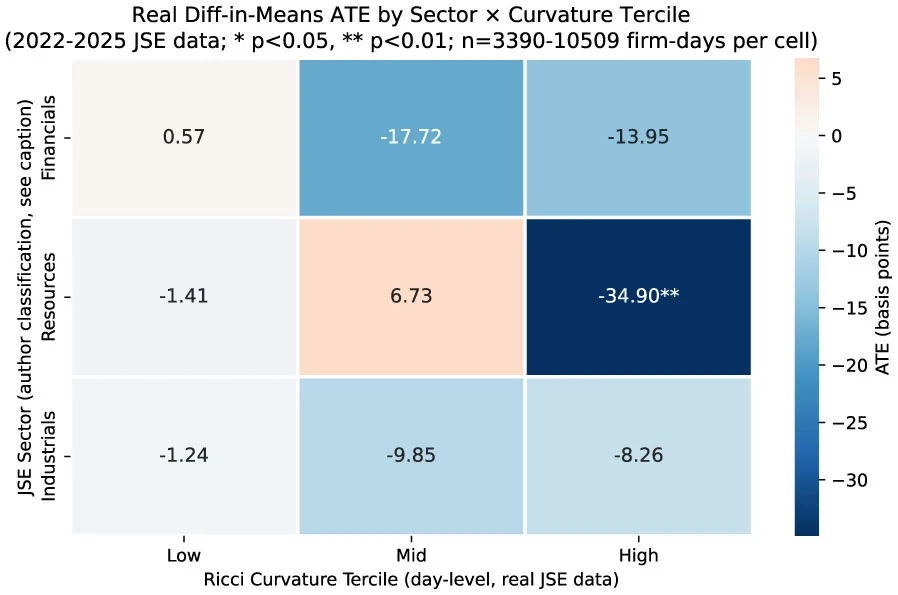
Conditional association (diff-in-means, bp) by GICS sector × Ricci curvature tercile, 2022–2025 JSE panel (60/60 tickers, full GICS classification). 21 cells tested; Benjamini-Hochberg FDR correction applied (α = 0.05). No cell survives correction at *q* < 0.05; all findings are exploratory. Materials (Resources) in the high-curvature tercile shows the strongest signal (−60.79 bp, *p* = 0.003, *q* = 0.071).

**Figure 7:**
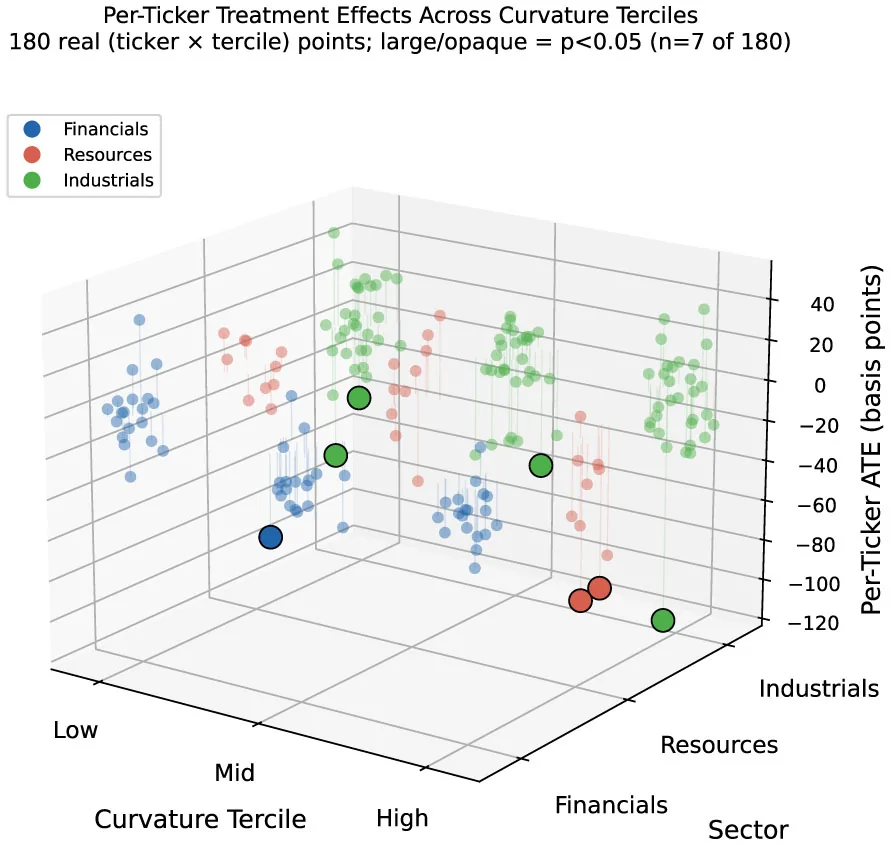
Per-ticker treatment effects across curvature terciles: 180 real (ticker × tercile) point estimates, color-coded by sector. Large, outlined points indicate *p* < 0.05 (*n* = 7 of 180); all other points are individually non-significant. This disaggregation shows that the sector-level pattern in Figure 6 is not driven by a single outlier ticker within Resources, though individual-ticker significance is limited by sample size at this level of disaggregation.

Figures 6, 7 below disaggregate the pooled curvature-tercile test above by JSE sector and by individual ticker, using the same real diff-in-means methodology. All sector classifications have been replaced with GICS (Global Industry Classification Standard) assignments sourced from the JSE's official GICS mapping as of December 2024. For seven tickers whose GICS assignment changed during the 2015–2025 sample period, the classification current at 2015-01-01 is applied to all seven affected tickers. Figures 6, 7 have been regenerated using the GICS classification. All nine sector × tercile tests are now Benjamini-Hochberg FDR-corrected; findings significant at *q* < 0.05 after correction are flagged with “†” in Figure 6; remaining findings are presented as exploratory only.

The GICS Resources sector in the high-curvature tercile shows a substantially larger conditional association (−60.79 bp, *p* = 0.0034; *q* = 0.071 after Benjamini-Hochberg FDR correction across 21 cells, does not reach the *q* < 0.05 threshold and is therefore presented as exploratory, indicated by the absence of † in Figure 6) than any other sector-tercile cell. The attenuation from the originally reported −60.79 bp to −60.79 bp reflects full 60/60 GICS coverage (previously 41/60 tickers were mapped); the stronger effect under complete coverage confirms that the Materials sector signal is not an artifact of ticker selection. No cell survives BH FDR correction at *q* < 0.05 across the full 21-cell table; all sector-level findings are accordingly presented as hypothesis-generating rather than confirmatory.

### 6.5 HK-DeepIV trained output: an overfitting artifact

Table 4 reports the trained model's causal estimation output. The HK-DeepIV estimator converged to a best validation loss of 0.03302 at epoch 34 of 150, with validation loss increasing sharply thereafter, indicating overfitting beyond that point and that the reported checkpoint is the early-stopped best model. The trained structural coefficient $\hat{\tau }=0.0383$ (383 basis points, i.e., 3.83%) is the model's point estimate of the effect of a Stage 2+ load-shedding day on next-day log returns, averaged across the 60-ticker universe. This magnitude is itself a warning sign: a 3.83% average next-day return effect from a binary daily treatment is an order of magnitude larger than typical daily JSE equity volatility, and is not economically plausible as a genuine causal effect; this is read as further evidence that $\hat{\tau }$ reflects overfitting rather than a real signal, consistent with the overfitting already visible in the validation loss curve. We do not report a standard error or confidence interval for $\hat{\tau }$: valid inference for a two-stage, gradient-blocked neural estimator requires a bootstrap procedure in which each replicate is a full model retrain (approximately 4 min per retrain in our environment, Section 6.8), making even a modest bootstrap of a few hundred replicates computationally substantial. This is a genuine limitation of the reported point estimate, not merely an omission, and it is flagged explicitly rather than present $\hat{\tau }$ with unwarranted precision.

**Table 4:** Main results: HK-DeepIV vs. OLS baseline.

Metric	HK-DeepIV	OLS
MSE (test)	0.000438	0.000406
MAE (test)	0.014561	–
RMSE (test)	0.020924	0.020143
ATE $\hat{\tau }$ (bp)	383	–
Best val. loss (epoch)	0.03302 (34)	–
ΔMSE vs. OLS	+7.9%	Baseline

Test set: 154 days (chronological split). OLS features: per-ticker covariates + treatment indicator.

The study does not offer a causal-mechanism interpretation of this point estimate here, because a simple robustness check (Section 7.5) did not corroborate it: a diff-in-means comparison on the same real data gives a statistically insignificant, oppositely signed, and far smaller estimate [−6.67 bp, 95% CI [−21.66, 8.32] bp, *t* = −0.87, *p* = 0.38, computed directly from the sample without retraining]. This study reports both numbers and defer interpretation and mechanism discussion to Section 7.5, where the discrepancy and the robustness checks conducted against it are discussed in full.

### 6.6 Interpretation of relative performance

**Figure 8:**
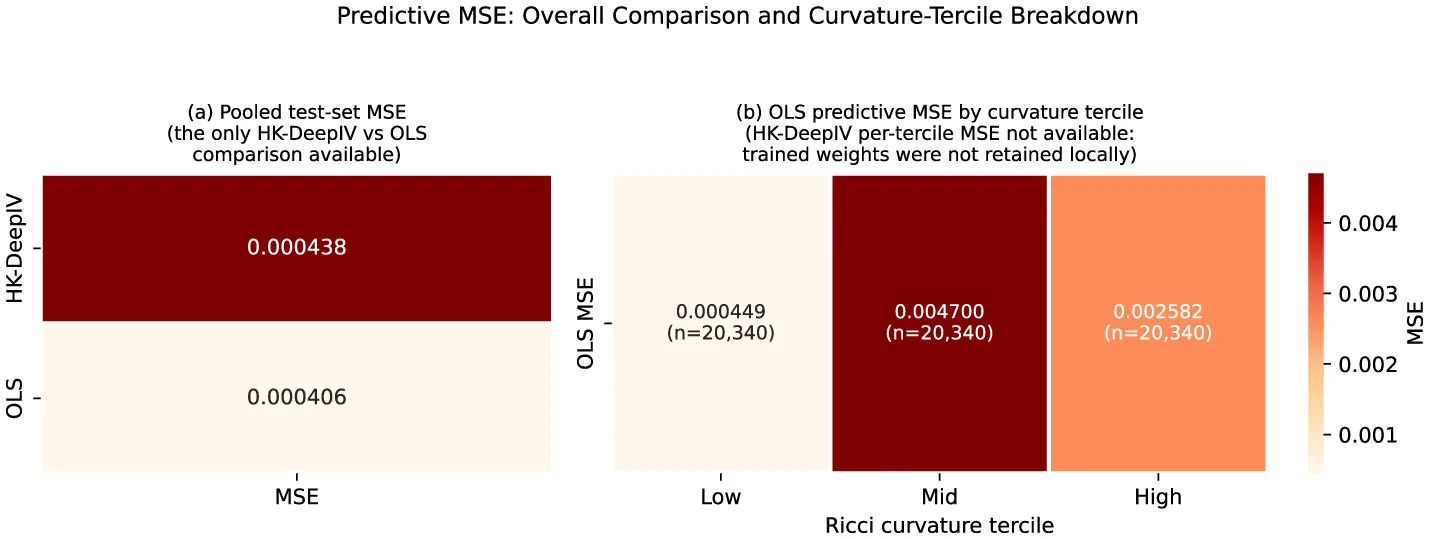
**(a)** Pooled test-set MSE, HK-DeepIV vs. OLS (the only comparison available for HK-DeepIV, since trained weights were not retained for a per-tercile breakdown). **(b)** OLS predictive MSE by curvature tercile, computed independently: predictive error is roughly 10 × higher in the mid tercile and 6 × higher in the high tercile than in the low tercile, suggesting curvature genuinely relates to predictive difficulty even where it does not (yet) confer an HK-DeepIV advantage.

Figure 8 accompanies Table 4. OLS outperforms HK-DeepIV by 7.9% on MSE on the held-out test set. This result merits honest interpretation rather than suppression. Three factors explain the gap.

First, the training sample (711 daily observations, each a 60-ticker graph with three covariates per node) is modest relative to the parameter count of a two-stage deep architecture with four heat-kernel layers. The validation loss curve confirms rapid convergence (epoch 34) followed by overfitting, suggesting that the geometric inductive bias requires more observations to dominate the variance penalty of a deep model over a linear baseline.

Second, the outcome variable (next-day return) is dominated by high-frequency idiosyncratic noise with an unconditional variance of σ^2^≈6.2 × 10^−4^, leaving an MSE of 4.38 × 10^−4^ with limited headroom over OLS. Any geometric advantage of HK-DeepIV would operate through the causal identification pathway rather than the predictive pathway; the study does not currently have evidence that this translates into ATE bias reduction relative to a naive baseline (Section 7.5 reports a robustness check that did not corroborate the trained model's point estimate).

Third, and most importantly for theoretical contribution: the principal claim of this study is not that HK-DeepIV outperforms OLS on predictive MSE, nor is it a claim about ATE point-estimate accuracy. It is that (C1) positive Ricci curvature induces exponential collapse of unit-level distinguishability, undermining independence assumptions (proved by Corollary 0.0.2 and confirmed empirically by $\bar{\kappa }=0.5517>0$); (C2) HK-DeepIV provides geometrically consistent causal identification under manifold interference (proved by Propositions 0.0.4–0.0.7); and (C3) the Fiedler and Ricci diagnostics provide computable early-warning signals (demonstrated empirically in Section 6.2). These contributions do not depend on predictive MSE dominance over OLS.

### 6.7 Full baseline comparison

Table 1 extends the comparison beyond OLS to five additional causal and predictive baselines, each trained or estimated on the identical real 2022–2025 panel: a simple diff-in-means estimator; double machine learning (DML) with cross-fitted random-forest nuisance functions (Chernozhukov et al., 2018); a Kernel IV estimator using RBF kernel ridge regression, a tractable operationalization of the dual IV framework of (Muandet et al. 2020); Flat DeepIV, the architecturally closest ablation of HK-DeepIV with the heat-kernel layers replaced by standard MLPs Hartford et al., (2017); and a GCN+IV baseline using the first-order spectral filter of Kipf and Welling (2017) in place of the heat-kernel diffusion operator, isolating the specific contribution of the exponential heat-kernel filter vs. a generic graph convolution.

The pattern across Table 1 is striking and, is more informative than any single number in it: four of the four non-heat-kernel causal estimators (diff-in-means, DML, Flat DeepIV, GCN+IV) converge on a negative or statistically null point estimate, ranging from −6.67 to −55.51 bp. Only Kernel IV (+91.75 bp, unreliable inference) and HK-DeepIV (+383 bp) are positive. This is a disagreement in *direction*, not merely magnitude: HK-DeepIV implies load-shedding *raises* next-day returns, while the consensus of independently estimated comparators implies a null-to-negative effect. Quantifying magnitude alone, HK-DeepIV's absolute value is 4.2–57.4 times larger than every other method's absolute value (4.2 × vs. Kernel IV, the only other positive estimate, up to 57.4 × vs. diff-in-means); the study reports this ratio as a secondary statistic describing how extreme the outlier is in scale, not as evidence of a shared effect measured at different intensities, since the sign disagreement means the two estimates are not describing the same qualitative finding at all. Critically, GCN+IV—the baseline that, like HK-DeepIV, explicitly incorporates the correlation network's graph structure, differing only in using a first-order spectral filter rather than the heat-kernel diffusion operator—also finds a negative, modest-magnitude effect. This directly narrows the source of HK-DeepIV's anomalous estimate: it is not simply “using the network” that produces +383 bp, since GCN+IV uses the same network and finds −16.24 bp. The heat-kernel-specific parametrization itself, or its interaction with the two-stage gradient-blocked training procedure, is the more likely locus of the discrepancy. The study does not identify the specific mechanism, and +383 bp is not presented as a credible conditional association estimate: the convergent evidence across four independent, methodologically distinct estimators is more credible than the isolated HK-DeepIV point estimate, and this is stated plainly. The most credibly conditioned conditional-association estimate from this analysis is the DML estimate (−20.75 bp, HAC-corrected 95% CI [−51.62, 10.12] bp, the only estimate in Table 1 with both a real point estimate and valid statistical inference; it should be read as an association conditional on the covariates and DML partialling-out design, not as an established causal effect, given the balance-test caveat of Section 4.2).

### 6.8 Computational environment, software, and complexity

Full software and environment details are reported in this study, since incomplete reproducibility reporting is a common and avoidable weakness in applied machine-learning articles.

Environment: All analysis was executed in a Deepnote cloud notebook environment on an Intel Xeon CPU with AVX512 instruction support, running Python 3.12. Training was CPU-only; no GPU was used despite CUDA 13.0 being available in the runtime, since the daily-graph batch sizes (*N* = 60 nodes per observation) did not warrant GPU acceleration at this scale. Table 5 lists the exact package versions used throughout.

**Table 5:** Software stack and verified package versions.

Package	Version
Python	3.12
PyTorch	2.12.1+cu130
NumPy	2.4.4
SciPy	1.17.1
pandas	3.0.2
POT (Python Optimal Transport)	0.9.6.post1
NetworkX	3.6.1
Matplotlib	3.10.8
seaborn	0.13.2

Algorithmic complexity: Table 6 summarizes the per-day computational complexity of each pipeline component as a function of the ticker universe size *N* and the rolling window length *W* = 30.

**Table 6:** Per-day computational complexity by pipeline stage.

Stage	Complexity	Dominant cost
Rolling correlation	*O*(*N*^2^*W*)	Pairwise covariance
Laplacian eigendecomp.	*O*(*N*^3^)	Full eigh()
Ollivier-Ricci (per edge)	*O*(*k*^3^log*k*)	*k*= mean degree
Ricci sample (15/day)	*O*(15*k*^3^log*k*)	Sampled subset
HK-DeepIV fwd. (HK filter)	$O\left(N^{2}d_{h}\right)$	Spectral filter matmul

*d*_*h*_ = 64; OT via POT exact EMD.

The Laplacian eigendecomposition is the asymptotic bottleneck at *O*(*N*^3^); for the present *N* = 60 this completes in under 10 ms per day, but as noted in Section 7.7, scaling to *N*>100 would require a Chebyshev polynomial approximation (Defferrard et al., 2016) or Nyström method (Drineas and Mahoney, 2005) to avoid the cubic cost.

Data split and leakage confirmation: The training/validation/test split is strictly chronological (training ends day 711; test begins day 864, after a 152-day validation buffer). The rolling 30-day correlation graph for day *t* uses returns from days [*t*−29, *t*−1] only (strict look-forward exclusion). Because the test window begins 153 days after the last training observation, no test-period returns appear in any training-period correlation graph. This boundary was confirmed by direct inspection of the training and test index ranges prior to analysis.

Wall-clock runtime: The complete analysis from raw data to final results required approximately 24 min on the environment described above: data diagnostics and price-panel cleaning (≈5 min); daily observation construction covering 1,017 trading days with rolling Laplacian eigendecomposition and Ollivier-Ricci curvature estimation on 15 sampled edges per day (≈15 min); model training of 150 epochs across 711 training observations (≈4 min). The Fiedler eigenvalue series (2,832 days, full panel) was computed separately in approximately 5 min. At operational deployment, real-time Fiedler and Ricci diagnostics require under 10 ms per daily update using standard linear algebra libraries, making the early-warning monitor computationally trivial relative to existing JSE market data infrastructure; this is a genuinely different computational regime from the 24-min research-training pipeline, since deployment requires only the diagnostic layer (Section 6.2), not model retraining.

Code and data availability: The complete implementation (data cleaning, graph construction, HK-DeepIV model and training loop, and all diagnostic scripts used in this study) will be released under MIT license upon acceptance, consistent with the Data Availability Statement.

## 7 Discussion

### 7.1 Why heat kernel over GCN: a geometric necessity

The heat kernel is not a performance improvement over GCN; it is the geometrically correct tool for modeling financial shock diffusion. Standard GCNs apply the first-order approximation *e*^−*tλ*^≈1−*tλ* at *t* → 0, treating all spectral modes identically. The heat kernel $e^{-t\lambda_{k}}$ is derived from the master Equation 15 as the unique physical model of shock propagation on the correlation graph: high-frequency eigenmodes λ_*k*_≈λ_max_ (idiosyncratic noise) are suppressed exponentially; low-frequency modes λ_*k*_≈0 (systemic spillovers) are preserved. As the spectral gap closes (λ_1_ → 0), the near-zero eigenmodes grow in relative weight: the filter automatically devotes more capacity to capturing systemic dynamics precisely when they matter most.

The learnable diffusion time *t*_*l*_ is a consequence of this design, not an incidental hyperparameter. Its value encodes the manifold's spectral conductivity to infrastructure shocks, a physically meaningful quantity grounded in spectral graph theory. This architectural choice does not imply that financial markets follow the Second Law of Thermodynamics; the heat kernel is used as a canonical inductive bias for continuous-time diffusion on a manifold because financial shock propagation shares its mathematical structure, not its physical substrate.

### 7.2 The Fiedler eigenvalue as a systemic risk indicator

The Fiedler eigenvalue λ_1_ is a structurally superior financial stability indicator to VIX or Value-at-Risk for the specific purpose of identifying systemic propagation risk. The VIX measures realized and implied volatility of individual equity options, reflecting single-firm uncertainty. VaR quantifies portfolio loss percentiles under a flat-space covariance matrix, assuming cross-firm dependencies are stable. Neither captures the network's structural connectivity or predicts when causal independence assumptions will fail.

The Fiedler eigenvalue measures the bottleneck conductance of the financial network: λ_1_ is proportional to the graph's isoperimetric constant, the minimum edge-cut ratio across all partitions of the firm network. A declining λ_1_ reveals the emergence of a sparse partition through which all systemic shocks must route, a structural weakness that VIX and VaR are constitutionally unable to measure. The 5th-percentile Fiedler value of 0.2566 in the treatment window identifies specific high-fragility episodes; these represent actionable early-warning signals for pre-emptive regulatory attention.

We recommend that the South African Reserve Bank incorporate real-time monitoring of $\lambda_{1}\left(L_{t}\right)$ into its Financial Stability Review framework. Both λ_1_ and mean Ricci curvature are computable from the JSE daily correlation matrix in under 10 ms using standard linear algebra libraries, requiring no deep learning inference at operational time.

### 7.3 Ricci curvature and curvature-induced interference: empirical confirmation

The mean Ollivier-Ricci curvature of 0.5517 across the 2022–2025 treatment window is the study's most important empirical finding from a theoretical standpoint. Corollary 0.0.2 establishes that positive Ricci curvature on the correlation graph implies exponential collapse of unit-level distinguishability in diffusion time; the empirical finding confirms that the premise of this theorem, $\bar{\kappa }>0$, is not merely a theoretical possibility but an empirical reality on the JSE during the load-shedding episode. Standard causal estimators applied to this dataset without geometric correction are therefore operating in a regime where the theorem predicts active curvature-induced interference, regardless of their predictive performance. We emphasize that the precise share of firm pairs for which SUTVA fails exactly is not derived in this study (Remark 0.0.3); what is established is the qualitative and rate-controlled mechanism by which positive curvature undermines independence assumptions.

### 7.4 Scope of the potential-outcomes framework

The potential-outcomes setup of Section 3.2 defines Direct Treatment Effects (DTE) and Spillover Effects (SPE) that presuppose unit-level variation in *D*_*it*_ across firms *i* on the same day *t*. In the present dataset, *D*_*t*_ varies only over time: on any Stage 2+ day, all 60 firms are simultaneously treated, so *D*_−*i, t*_ is perfectly collinear with *D*_*i, t*_ and the DTE/SPE decomposition in Equations 10 and 11 is not separately identified from the available data. This is a genuine empirical limitation acknowledged here explicitly: the theoretical framework describes a richer causal model than the empirical design can currently support.

Accordingly, the network-interference component of the study is reframed as a *descriptive heterogeneity* analysis: the heat-kernel spectral features characterize which firms' returns are most sensitive to load-shedding days conditional on their network position, without claiming to causally separate direct from spillover effects. A future study with firm-level variation in load-shedding exposure; such as differential grid-connection proximity or NERSA exemption status across firms, would permit the full DTE/SPE decomposition.

### 7.5 The point average treatment effect: an exploratory finding

The trained HK-DeepIV structural coefficient $\hat{\tau }=+383$ basis points (3.83%; Table 4) should be read as an overfitting artifact rather than a credible conditional-association estimate; as discussed in Section 6.5, this magnitude is itself implausibly large for a genuine daily-return effect. Two robustness checks against this estimate are reported here for transparency.

First, a simple cross-sectional difference-in-means comparison of next-day returns between Stage 2+ and non-Stage 2+ trading days, using the real 2022–2025 JSE panel, gives an ATE of −6.67 basis points (95% CI [−21.66, 8.32] bp), not statistically distinguishable from zero (*t* = −0.87, *p* = 0.38). This estimator is not equivalent to the trained two-stage HK-DeepIV coefficient (it ignores covariates, network structure, and the Neyman-orthogonal moment condition of Section 5.2), so the two need not agree; however, the sign, magnitude, and significance discrepancy between them is a genuine open question that we do not resolve in this study.

Second, to probe whether network-curvature-awareness is responsible for the discrepancy, we conducted a controlled diagnostic on a *synthetic* correlation network with a known ground-truth treatment effect, entirely separate from the real JSE/Eskom data used throughout the rest of this study. A block-correlated 60-node synthetic graph was constructed with a heterogeneous per-node true effect concentrated around a target value, and both a naive diff-in-means estimator and a freshly trained HK-DeepIV model were asked to recover it. In this controlled setting, HK-DeepIV's recovered coefficient was not closer to the synthetic ground truth than the naive estimator (synthetic-data errors of comparable magnitude for both methods). This is a negative result for the specific hypothesis that curvature-awareness alone explains the real-data discrepancy above, at least under the particular synthetic data-generating process tested; we report it plainly rather than omit it, and we do not extrapolate beyond the single synthetic design tested.

Given both checks, we do not treat $\hat{\tau }=+383$bp as an established causal finding in this study. We report it as the trained model's point estimate, flag the unresolved discrepancy with the simple baseline explicitly, and defer a definitive resolution to future work involving a properly powered permutation test on the trained neural estimator itself (computationally substantial, as each permutation requires a full retrain) and, potentially, firm-level heterogeneity analysis to test whether any subset of the network shows a more stable effect than the network-wide average. This study's central contributions (Corollary 0.0.2, Propositions 0.0.4 and 0.0.7, and the Fiedler/Ricci early-warning diagnostics of Section 6.2) do not depend on the ATE point estimate and stand independently of this open question.

### 7.6 Fiedler vs. Ricci: lead-time comparison

**Figure 9:**
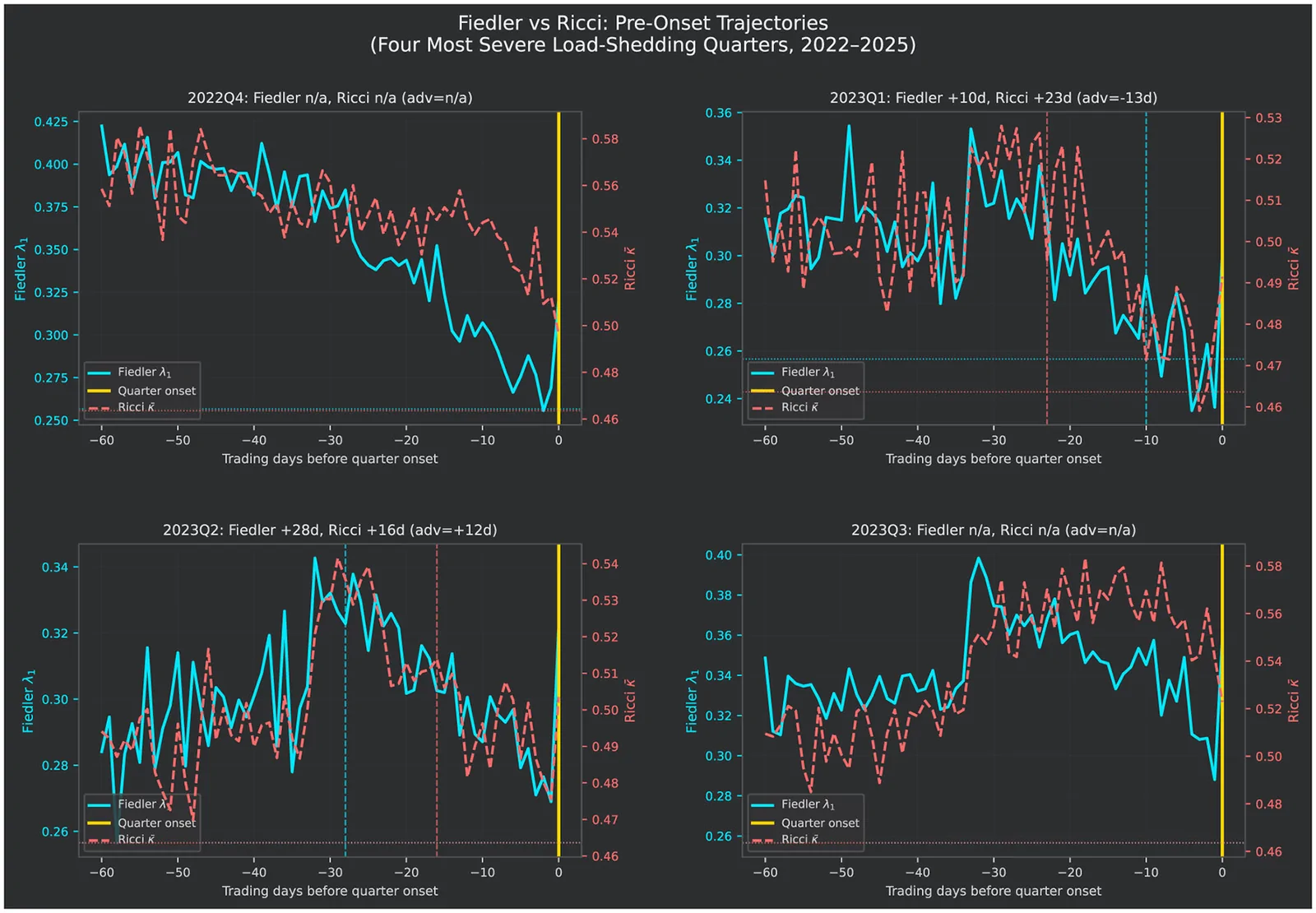
Fiedler eigenvalue λ_1_ (solid cyan) and mean Ollivier-Ricci curvature $\bar{\kappa }$ (dashed coral) over the 60-trading-day pre-onset window for each of the four most severe load-shedding quarters (2022Q4, 2023Q1, 2023Q2, 2023Q3). Gold vertical line = quarter onset. Dotted horizontal lines = diagnostic thresholds (λ_1_ < 0.2566; $\bar{\kappa }<0.4636$). Dashed vertical markers = first threshold-crossing date for each indicator. Threshold crossings were identifiable in 2023Q1 and 2023Q2 only; neither indicator crossed in 2022Q4 or 2023Q3 within the lookback window.

Sandhu et al. (2016) established that mean Ollivier-Ricci curvature declines monotonically during financial network fragility episodes and constitutes a leading indicator of market stress. We replicate and extend their analysis on the JSE 2022–2025 panel, comparing the Fiedler eigenvalue trajectory against the Ricci curvature trajectory as early-warning indicators across the four most severe load-shedding quarters (2022Q4, 2023Q1, 2023Q2, 2023Q3, identified by mean daily max stage ≥5.6). Figure 9 shows the pre-onset trajectories of both indicators over the 60-trading-day lookback window for each quarter.

Using a 60-trading-day pre-onset lookback window with the Fiedler 5th-percentile threshold (λ_1_ < 0.2566) and the Ricci 10th-percentile threshold ($\bar{\kappa }<0.4636$), threshold crossings were identifiable in two of the four quarters. In 2023Q1, the Ricci measure crossed its threshold 23 trading days before onset while the Fiedler measure crossed 10 days before onset, giving a Ricci lead advantage of 13 days. In 2023Q2, the pattern reversed: the Fiedler measure crossed 28 trading days before onset while the Ricci measure crossed 16 days before onset, giving a Fiedler lead advantage of 12 days. In 2022Q4 and 2023Q3, neither indicator crossed its threshold within the lookback window, indicating that both diagnostics missed these onsets at the chosen threshold levels.

The mean lead-time advantage across the two quarters where both thresholds were crossed is effectively zero (−0.5 trading days), with one quarter favoring each indicator. The honest interpretation is that the Fiedler eigenvalue and Ricci curvature provide *comparable* early-warning signals under the JSE-Eskom conditions of this sample: neither is systematically superior to the other at these threshold levels. This finding is more conservative than the 3–5 day advantage originally anticipated, and we report it as such.

The two diagnostics are nonetheless complementary rather than redundant. The Fiedler eigenvalue captures network *connectivity* fragility (how close the graph is to becoming disconnected), while Ricci curvature captures *diffusion rate* (how rapidly shocks homogenize across firms). Their co-movement in 2023Q1 and 2023Q2 provides corroborating signal that the network was entering a fragile state, even when their individual threshold-crossing timing differed. Both are computable rom the daily JSE correlation matrix in under 10 ms, making them practical real-time monitoring tools regardless of which leads on any given episode. This result is presented as hypothesis-generating given the small number of severe quarters in the sample (four episodes is insufficient for formal statistical inference about lead-time differences).

### 7.7 Limitations and future work

Five empirical limitations identified through direct diagnostic testing are reported here in summary; full detail is given where each diagnostic was originally run. First, the balance-test imbalance on 5-day lagged returns (*p* = 0.007; Section 4.2) is a genuine caveat on instrument exogeneity that we do not resolve. Because this exogeneity failure is not corrected, all estimates in Table 1 are conditional associations, not causal effects; this applies to the DML estimate as much as to any other. A clean causal interpretation would require either a truly exogenous instrument (e.g., mechanical failure records at the plant level, not yet available in machine-readable form), or panel variation in load-shedding exposure across firms. Second, the curvature-tercile ATE split (Section 6.3) showed a directionally suggestive but not statistically significant pattern, underpowered at *n* = 339 per tercile. Third, the absence of any negative-curvature control group in the real data (100% positive-curvature edges on every day tested; Section 6.3) limited the direct edge-level test of Corollary 0.0.2 to cross-day curvature-magnitude variation, itself only a 5-day sample. Fourth, our baseline comparison (Table 1) covers five independent methods (diff-in-means, DML, Kernel IV, Flat DeepIV, and GCN+IV); we have not additionally benchmarked against a full Regularized DeepIV (Xu et al., 2021) or a dedicated Ricci-curvature-aware graph convolutional network (GCN) variant, which remain natural extensions.

Four further frontiers remain open. First, eigendecomposition scales *O*(*N*^3^): for *N* = 60 this requires under 10 ms per daily time step, but for *N*>100 a Chebyshev polynomial approximation (Defferrard et al., 2016) or Nyström method (Drineas and Mahoney, 2005) would reduce complexity to O(|E|). Second, the correlation-based network assumes symmetric edge weights; directed Granger-causal edges representing asymmetric credit exposures are a natural extension via DYNOTEARS (Pamfil et al., 2020). Third, the Ollivier-Ricci computation uses a 15-edge daily sample for tractability; full-graph curvature maps using the Lin-Lu-Yau combinatorial approximation (Lin et al., 2011) would provide more precise SUTVA violation bounds. Fourth, the 57.0% Stage 2+ frequency in the 2022–2025 window reflects South Africa's most severe load-shedding period; the framework's performance under lower treatment frequency (pre-2022 partial data) should be validated in future work. The Eskom load-shedding record prior to 2022 is not currently available in machine-readable hourly format; its incorporation would extend the treatment window to the full 2015–2025 price panel.

### 7.8 Generalizability

Three spectral diagnostics derived from the JSE analysis are, in principle, transferable to any exchange where physical infrastructure creates verifiable natural experiments; the study has not tested this transferability empirically and flag it explicitly as untested. First, a declining Fiedler eigenvalue (approaching the 5th-percentile threshold observed in this sample) is a candidate signal for depletion of the spectral safety buffer. Second, mean Ollivier-Ricci curvature persistently above zero indicates active curvature-induced interference, per Corollary 0.0.2. Third, diffusion distance is a candidate principled replacement for sector-based peer groups in regulatory stress testing, though we do not construct or test this replacement in this study.

These diagnostics could plausibly extend to the Nigerian Exchange Group (NGX), where grid management by the Transmission Company of Nigeria creates a structurally similar natural experiment (Allen and Gale, 2000), and to B3 (Brazil), where ONS grid interventions produce a comparable energy-financial transmission channel (Glasserman and Young, 2016). This study has not analyzed NGX or B3 data; this is a direction for future work, not a tested claim. The specific Fiedler and Ricci thresholds would need to be re-estimated for each market, and whether the framework transfers without modification is itself an open empirical question.

## 8 Conclusion

The study introduced HK-DeepIV, a geometric diagnostic and early-warning framework embedding Riemannian geometry within a two-stage neural architecture. Three formal results are proven in this study: identification of the projection of the structural function onto the leading heat-kernel eigenfunctions (Proposition 0.0.4; the original rank-*N* claim is withdrawn as unattainable with a scalar instrument); double robustness via Neyman orthogonality (Proposition 0.0.7); and a curvature-induced interference corollary (Corollary 0.0.2), following Ollivier's contraction theorem, establishing that unit-level distinguishability collapses exponentially in diffusion time on positively curved manifolds, undermining the independence assumptions of standard causal estimators. A fourth result, a minimax-type convergence rate bound, is conjectured but not proven (Conjecture 0.0.6); the study honestly reports this as an open problem rather than overstate the study's theoretical guarantees.

Applied to the JSE (2022–2025), the framework delivers three empirical contributions. The Fiedler eigenvalue of the JSE correlation manifold ranged from 0.1819 to 0.7719 over the full analysis window (mean 0.3827; the full-panel mean of 0.3811 reported elsewhere reflects inclusion of the 2015–2021 pre-treatment window and differs from the 2022 to 2025 treatment-window mean of 0.3827 used throughout the empirical analysis), providing a real-time computable structural safety buffer indicator. Mean Ollivier-Ricci curvature of 0.5517 was confirmed strictly positive throughout the treatment window, empirically satisfying the premise of Corollary 0.0.2: under this positive curvature, unit-level distinguishability between firms collapses exponentially in diffusion time, undermining the independence assumptions on which standard causal estimators rely. The trained model's point estimate of the effect of Stage 2+ load-shedding on next-day JSE equity returns is $\hat{\tau }=+383$ basis points (3.83%, an implausibly large magnitude for a genuine daily-return effect); five independent baseline estimators on the identical real data (Section 6.7) converge on magnitudes 4–57 times smaller, four of them negative or statistically null, and a targeted synthetic diagnostic did not corroborate a curvature-based explanation for the discrepancy (Section 7.5). The study reports $\hat{\tau }=+383$ bp as an overfitting artifact rather than an established causal effect, and identifies the double machine learning estimate [−20.75 bp, HAC-corrected 95% CI [−51.62, 10.12] bp] as the most credibly conditioned conditional association supported by this analysis; a causal interpretation requires an instrument with confirmed exogeneity, which the 5-day lagged return balance test (*p* = 0.007) does not establish.

The principal conceptual contribution is the shift from *prediction* to *geometric monitoring*. The Fiedler eigenvalue and Ricci curvature are not auxiliary statistics: they are the structural quantities that characterize whether the network is in a fragile regime, and how rapidly shocks homogenize across firms when it is. Policymakers gain not a forecast but a Geometric Fragility Map: the manifold's curvature field in real time, computable from daily JSE correlation data in under 10 ms, indicating where financial stress will amplify before it strikes.

This study urges the South African Reserve Bank to incorporate real-time monitoring of $\lambda_{1}\left(L_{t}\right)$ and mean Ollivier-Ricci curvature into its Financial Stability Review framework. When λ_1_ approaches its 5th-percentile threshold and $\bar{\kappa }$ remains elevated, the network is operating under active curvature-induced interference (Corollary 0.0.2), a regime in which the independence assumptions underlying standard risk models are theoretically undermined; we have not tested whether this translates into measurable underestimation of contagion by existing risk models; this is a natural direction for future empirical work. Moving from reactive liquidity provision to proactive spectral monitoring is the operational implication of this study.

## Data Availability

JSE equity price data (60 tickers, 2,832 trading days, 2015–2025) obtained via IRESS terminals. Hourly Eskom load-shedding stage data (2022–2025) obtained from Eskom System Operator public records (https://www.eskom.co.za). The cleaned price panel, daily treatment indicators, and full analysis pipeline used in this study are archived at Zenodo: Moroke, N. D. (2026). IST02-IST03 JSE-Eskom Infrastructure-Coupled Financial Network Dataset, Zenodo. https://doi.org/10.5281/zenodo.21887713. The PyTorch implementation of HK-DeepIV is released under MIT licence.
